# A versatile microfluidic device for multiple ex vivo/in vitro tissue assays unrestrained from tissue topography

**DOI:** 10.1038/s41378-020-0156-0

**Published:** 2020-06-29

**Authors:** Jose M. de Hoyos-Vega, Alan M. Gonzalez-Suarez, Jose L. Garcia-Cordero

**Affiliations:** Unidad Monterrey, Centro de Investigación y de Estudios Avanzados del IPN, Via del conocimiento 201, Parque PIIT, Apodaca, NL 66628 Mexico

**Keywords:** Electrical and electronic engineering, Chemistry

## Abstract

Precision-cut tissue slices are an important in vitro system to study organ function because they preserve most of the native cellular microenvironments of organs, including complex intercellular connections. However, during sample manipulation or slicing, some of the natural surface topology and structure of these tissues is lost or damaged. Here, we introduce a microfluidic platform to perform multiple assays on the surface of a tissue section, unhindered by surface topography. The device consists of a valve on one side and eight open microchannels located on the opposite side, with the tissue section sandwiched between these two structures. When the valve is actuated, eight independent microfluidic channels are formed over a tissue section. This strategy prevents cross-contamination when performing assays and enables parallelization. Using irregular tissues such as an aorta, we conducted multiple in vitro and ex vivo assays on tissue sections, including short-term culturing, a drug toxicity assay, a fluorescence immunohistochemistry staining assay, and an immune cell assay, in which we observed the interaction of neutrophils with lipopolysaccharide (LPS)-stimulated endothelium. Our microfluidic platform can be employed in other disciplines, such as tissue physiology and pathophysiology, morphogenesis, drug toxicity and efficiency, metabolism studies, and diagnostics, enabling the conduction of several assays with a single biopsy sample.

## Microsystems & Nanoengineering Microfluidics: Surface tissue assays

A microfluidic device enables eight independent assays to be performed simultaneously on the surface of topologically irregular tissue sections. Current limitations for assays of tissue slices include, typically, a limited amount of tissue sample—limiting the range of assays that can be performed for a uniform sample—and sensitivity to the surface of the tissue, which might be inconsistent due to the spatially irregular composition of tissue. Here, a team from the Center for Research and Advanced Studies of the National Polytechnic Institute, Mexico, led by Jose Garcia-Cordero, has developed a microfluidic device that can perform assays on tissue sections with a wide range of topologies and thickness. The device consists of eight independent channels, enabling parallelization, and is demonstrated for tissue from an aorta, for both in vitro and ex vivo assays.

## Introduction

Tissue slices are sections of tissues harvested in vivo, excised, sliced, explanted and cultured in vitro. Tissue slices were first introduced in 1923, but it was not until 1980 that interest was revived with the invention of a slicer that allowed tissues with a defined thickness and uniformity to be prepared (precision-cut tissue slices)^1^. Slices are commonly obtained with a vibratome, microtome or a tissue chopper^2^ but can also be obtained manually with a scalpel^3^ or a needle puncher^4^. Section thicknesses can range from 4 µm to 2 mm^5,6^, although the most common thickness for ex vivo studies ranges from 100 to 400 µm^7,8^. Tissue-cut slices resemble the in vivo histology of an organ because they retain organ cytoarchitecture (to a certain extent), contain the different types of cells that make up an organ, maintain organ matrix configuration^2,3,9^ and preserve metabolic enzymes, transporters, and cofactors^10^. In some cases, tissue slices have been shown to better predict in vivo metabolite profiles than cell lines and can quantitatively predict drug metabolite clearance^3^.

**Fig. 1:**
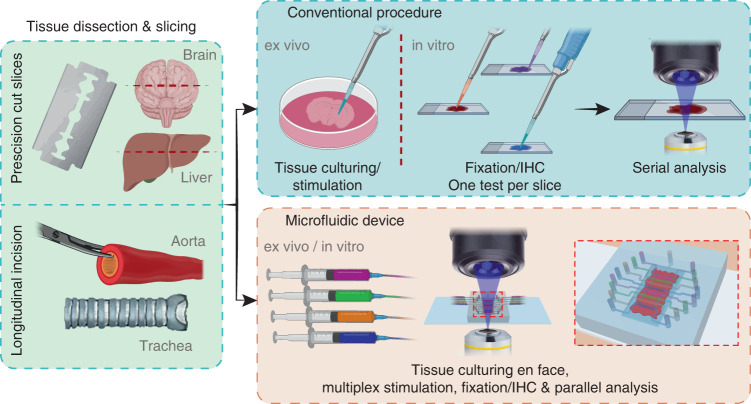
Comparison of the conventional procedure and our microfluidic device to analyze tissue slices. Top panel: Dissected tissue samples are sliced and cultivated in a static environment with a single stimulation. Samples are later fixed and stained on a glass slide for downstream serial analysis. Bottom panel: Tissue samples are placed in the microfluidic device, allowing for a dynamic culturing environment, multiple parallel stimulation, immunohistochemical (IHC) staining, and analysis

Classic techniques for organotypic slice culture include roller tube cultures, membrane cultures, and petri dishes^2^. Tissue-cut slices from the liver, kidney, lung, brain, glands, heart, prostate, and spleen have been used in a wide range of applications including drug metabolism and discovery, physiology, morphology, development, endocrinology, and toxicology^2,9,10^. Most tissue slices can be cultured for up to 24 h; however, using dynamic organ culture incubation systems with appropriate culture media, oxygen supply conditions, and media perfusion, this period can be extended for up to 7 days^3^ or even weeks to months^2^. Slices of different organs are prepared using similar straightforward techniques^10^. However, a major limitation of these techniques is that they require a single tissue sample for each biochemical assay, increasing the demand for samples that may be scarce (e.g., biopsies), notwithstanding the large volumes of media and reagents used^11–15^ (Fig. 1). Therefore, new technologies that perform multiple assays with a single tissue section will have tremendous impact on the fields of pharmacology, toxicology, tissue physiology, and pathophysiology, and diagnostics^9^. Ultimately, tissue slices can become an important tool to test drug efficacy, safety, and toxicology in humans before clinical trials.

Tissues are surrounded by connective tissue that has a key role in providing structure, repair, homeostasis, extracellular matrix production, nutrient supply, cell signaling and cell proliferation^16,17^. The removal of connective tissue alters ex vivo cellular responses. However, the connective tissue has a highly irregular composition: a variable thickness, an uneven surface, and different stiffness, cellular composition, and shape. These features hinder tissue analysis and are commonly removed to provide a smooth, flat tissue surface^7,13^. Furthermore, slicing tissues with a vibratome damages the outer layers of the tissue due to mechanical stress, requiring at least 1 h of incubation before an assay can be performed^18^. Thus, it is important to preserve the connective tissue to obtain a physiological response closer to in vivo.

Culturing tissue sections with irregular surfaces has been attempted in microfluidic devices, however, the tissue irregularities quickly lead to high reagent diffusion and cross-contamination^13,18–21^. Other microfluidic devices (Sup Table 1) have been reported to analyze tissue sections from different organs^6,7,22,23^, but they use either flat and homogenous tissue sections or fixed thin tissue slices of ~5 µm^5,24,25^, which renders them impractical to analyze tissue sections with irregular topographies. Thus, there is a need to develop technologies to study tissue sections ex vivo unrestrained by the physical and mechanical features of tissue sections, and versatile enough to perform multiple ex vivo and in vitro assays on a single tissue sample. These assays include fixing, staining, drug screening, introducing cells for immune studies, and evaluation of environmental risk factors, among others.

To address some of these issues, we developed a microfluidic platform that can accommodate tissue sections of varying topologies, thicknesses and organ sources. The device can perform multiple ex vivo and in vitro assays on a fresh tissue sample en face. To demonstrate the versatility of our platform, we carried out a drug-response assay, a viability assay, immunohistochemistry staining, and tissue-immune cell interaction studies. In the latter study, we demonstrated the interaction of neutrophils with the endothelia of a tissue section after stimulation with lipopolysaccharide (LPS).

## Results and discussion

### Tissue characteristics

**Fig. 2:**
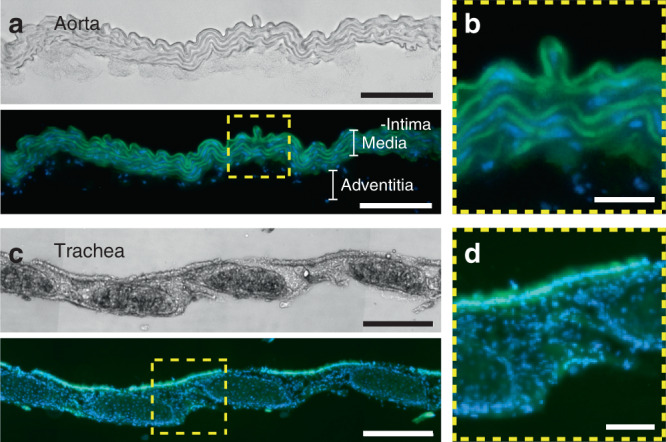
Tissue characteristics of the aorta and trachea. **a** Brightfield and fluorescence images of a 12 µm cryostat slice of the descending aorta en face showing an irregular surface and thickness profile. The tunica intima is located on the upper side of the tissue and lays on top of the tunica media composed of a thick variable layer of muscle fibers (autofluorescence). The adventitia layer can be distinguished by the scattered cells (blue, Hoechst staining) beneath the tunica media. Scale bar: left, 100 µm. **b** Magnification of the yellow square in (**a**). Scale bar: 25 µm. **c** Murine trachea section. Collagen rings (disk-like structures) and annular ligament segments can be identified in brightfield and fluorescence images of a murine trachea slice en face. The epithelial monolayer (green autofluorescence) is located on the top of the tissue. Scale bar: 200 µm. **d** Magnification of the yellow square in (**c**). Scale bar: 50 µm

To demonstrate the highly irregular topography of some tissues, micrographs (Fig. 2) of two irregular tissue sections used in this study, the murine aorta and trachea, are shown. The cross-sectional thickness of a murine aorta can range from 44 to 111 µm with an average of 74 µm, while the trachea thickness ranges from 76 to 171 µm with an average of 127 µm. The aorta, with its tunica intima, media and adventitia, represents a tissue with a uniform composition but with substantial thickness variability (Fig. 2a, b). The trachea, composed of interspersed collagen rings and annular ligaments, represents an irregular tissue morphology due to its interleaved stiff and soft sections along its length (Fig. 2c, d). It is clear from these micrographs that their topography is highly irregular and very different from the flat and homogeneous sections that are commonly used in tissue section studies. Thus, the challenge for any technology is to accommodate tissue sections with variations in height and differences in stiffness.

### Device design and fabrication

**Fig. 3:**
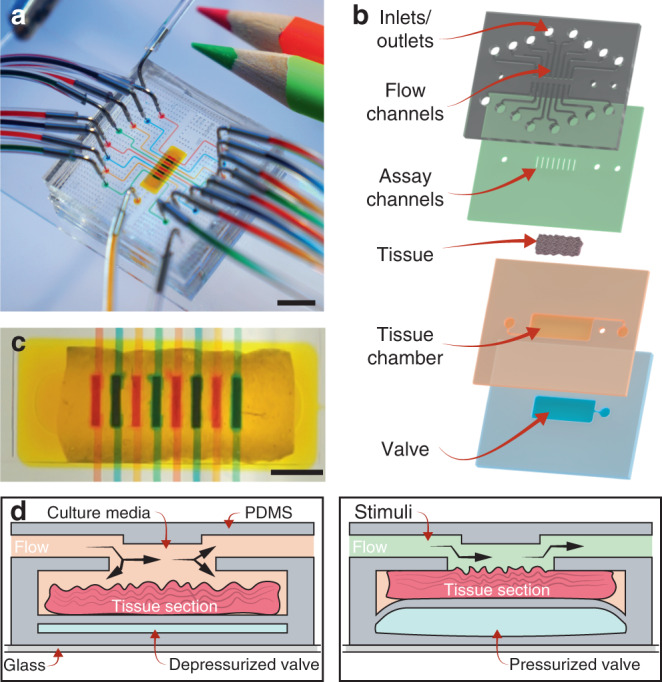
Device design and working principle. **a** Photograph of the microfluidic platform*;* scale bar: 5 mm. **b** Schematic of the assembly of the microfluidic device. The device is composed of four layers: (1) flow channels and eight inlets with their respective mirrored outlets; (2) 8 assay microchannels; (3) an open tissue chamber with an inlet and outlet; and (4) a valve. **c** Micrograph of a tissue section (an aorta in this case) inside the device with each assay microchannel independently perfused with different food dyes; scale bar: 1 mm. **d** Schematic cross-sectional view of the working mechanism of the microfluidic device. The left schematic illustrates a tissue section placed on the tissue chamber with an unpressurized valve in which media flows into the tissue chamber. When the valve is actuated (right schematic), the tissue section moves up and is sealed against the assay channels, creating closed channels, where only the top surface tissue is exposed to a stimulus

To work with these irregular tissue sections and perform multiple assays with an entire tissue excision, we developed a multilayer polydimethylsiloxane (PDMS) microfluidic device (Fig. 3a). The device consists of four stacked layers initially separated into two assemblies (Figs. 3b, S1). The bottom assembly comprises a valve located below an open tissue chamber, while the top assembly contains an array of eight assay channels (150-µm deep, 200-µm wide, and 1.1-mm long) that connect to an array of top open microchannels with eight inlets and outlets. A tissue section is manually placed in the tissue chamber before sealing with the top assembly (Fig. 3c). The tissue chamber features an outlet and an inlet, through which fresh media is introduced to constantly periperfuse the tissue. Drugs and solutions are introduced through the eight channel inlets.

Based on the dimensions of the open folded thoracic descending aorta (length: ~6 mm, width: ~2.5 mm), the tissue chamber was designed to be 7.6 mm × 2.9 mm. This additional room facilitated tissue deposition. (Note that the chamber dimensions and assay microchannels can be modified according to tissue and experimental needs.) To avoid contact of the tissue and liquid with the top layer before irreversible bonding, the tissue chamber was designed to be 100 µm taller than the thickest feature of the tissue (250-µm deep).

Located beneath the tissue chamber is a flexible valve with similar dimensions to the tissue chamber (Fig. 3b). When the valve is actuated, it pushes the tissue section upwards until it touches the assay microchannels, thus effectively creating enclosed channels separated from each other by 300 µm (Fig. 3d, c). This arrangement enables tissue culture and experimentation under eight different conditions at the same time. Other microfluidic devices for multiplexing ex vivo studies use open chambers that contain a porous membrane where the tissue section is placed or are assembled from different materials to encapsulate the tissue in the device^4,12,13,19,22,24^. In contrast to these approaches, our platform integrates all components in a monolithic PDMS device.

### Device characterization

We first characterized the surface profile that the valve acquires at different pressures. In its native state, the valve is flat, but when actuated, it resembles an arch that can reach up to 630 µm at its apex (Fig. S2). Because of this arch shape, the degree of tissue penetration in each of the eight channels is related to the position of the microchannel in the chamber and to the pressure applied on the valve (Fig. S3a–d). Confocal micrographs taken of the middle channel, actuated at 7 and 35 kPa, show that the tissue indeed penetrates the channel in a parabolic form (Fig. S3c). It is important to consider these cross-sectional area variations along the channel, as this variation will give rise to differences in flow velocity and can affect assays that require the precise control of flow velocity (Fig. S4). In general, we found that there was good sealing between the tissue and the channels with the valve actuated at more than 7 kPa. Importantly, we did not observe any delamination even at 35 kPa.

**Fig. 4:**
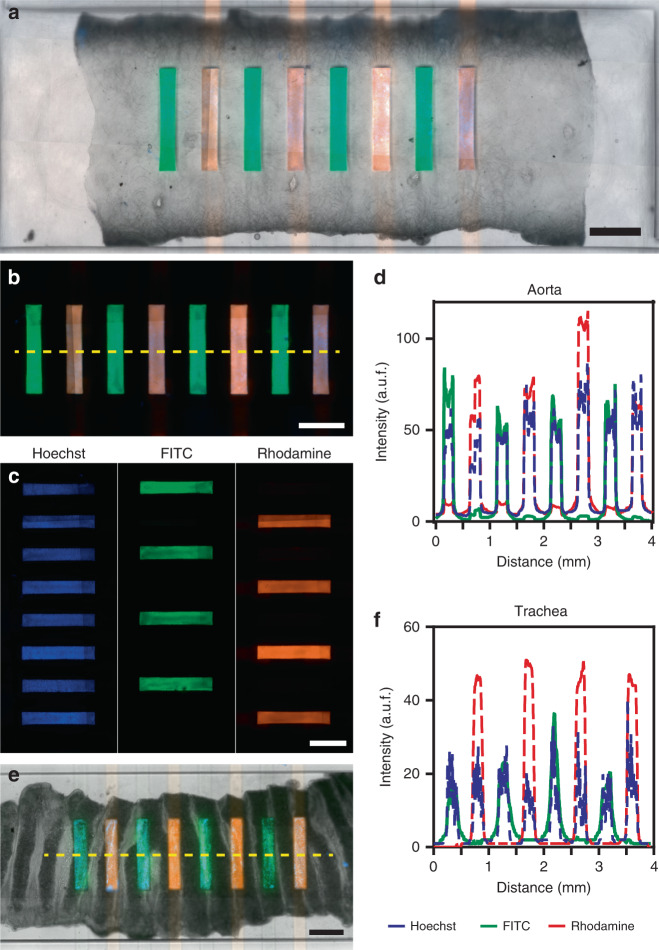
Leakage and intercellular diffusion assay on a tissue section. **a** Brightfield and fluorescence micrograph of an aorta in the tissue chamber when Dextran-R or FITC was flowed in the eight microchannels in combination with Hoechst; scale bar: 1 mm. **b** Fluorescence micrograph showing that fluorescence dyes are confined inside the assay microchannels; scale bar: 500 µm. **c** Micrographs of the separate fluorescence channels; scale bar: 500 µm. **d** Fluorescence intensity profile of the dotted line shown in image (**b**). **e** Merged brightfield and fluorescence micrograph of the trachea en face; scale bar: 500 µm. **f** Fluorescence intensity profile of the dotted line shown in image (**e**)

Next, we investigated whether there was any crosstalk between the microfluidic channels or biomolecule diffusion through the tissue from one channel into neighboring channels. These experiments were carried out at 16 kPa, which is the pressure that aortic tissues experience under normal physiological conditions^26^. We chose three fluorescent dyes of different molecular weights representing a range of biomolecules delivered into tissues^27,28^: two cell-impermeable dyes, dextran-rhodamine (40 KDa) and fluorescein isothiocyanate (389 Da), and a cell-permeable DNA staining dye, Hoechst (616 Da). While Hoechst flowed in all channels, the other two dyes flowed interspersed in the eight microchannels of the device. After 10 min of perfusion, neither dextran-rhodamine (Dextran-R) nor fluorescein isothiocyanate (FITC) leaked into adjoining microchannels, while Hoechst stained all the nuclei of the cells residing inside the channels (Fig. 4a–c). In comparison, in other microfluidic devices, leakage and intercellular diffusion of these biomolecules occurs in the first 10 s of perfusion, at least for brain and lymph node precision-cut slices^13,18,21^.

The fluorescent traces of the three dyes along the eight microchannels had similar profiles: a sharp increase in fluorescence intensity at the edges of the microchannels and a high level of fluorescence inside the width of the channel (Fig. 4d). In contrast, between the microchannels, the fluorescence levels were close to zero, indicating very little leakage. As shown in Figs. S5 and S6, only the cells inside the channels were stained. When a soft tissue is compressed, fluid in the interstitial space is expelled, preventing molecules from diffusing through these compressed regions. The solute diffusivity through a tissue depends on its physical and chemical properties and on the composition of the extracellular matrix^29^. These results demonstrated the ability of our device to confine the biomolecules inside the channels with no crosstalk between channels.

We also investigated the performance of our device with tissues of different stiffnesses, such as the trachea, which has softer regions. We obtained comparable fluorescence intensity profiles except for FITC, which leaked marginally in areas where the cartilage rings and annular ligaments converged at the edge of the microchannel walls (Fig. 4e, f). A possible solution to this leakage problem is to space the channels further apart and reduce the width of the microchannels to decrease the contact area of the tissue with the open microchannel. Further validation of our device should be carried out with tissue sections from other organs.

### Ex vivo experimentation

#### Tissue viability

**Fig. 5:**
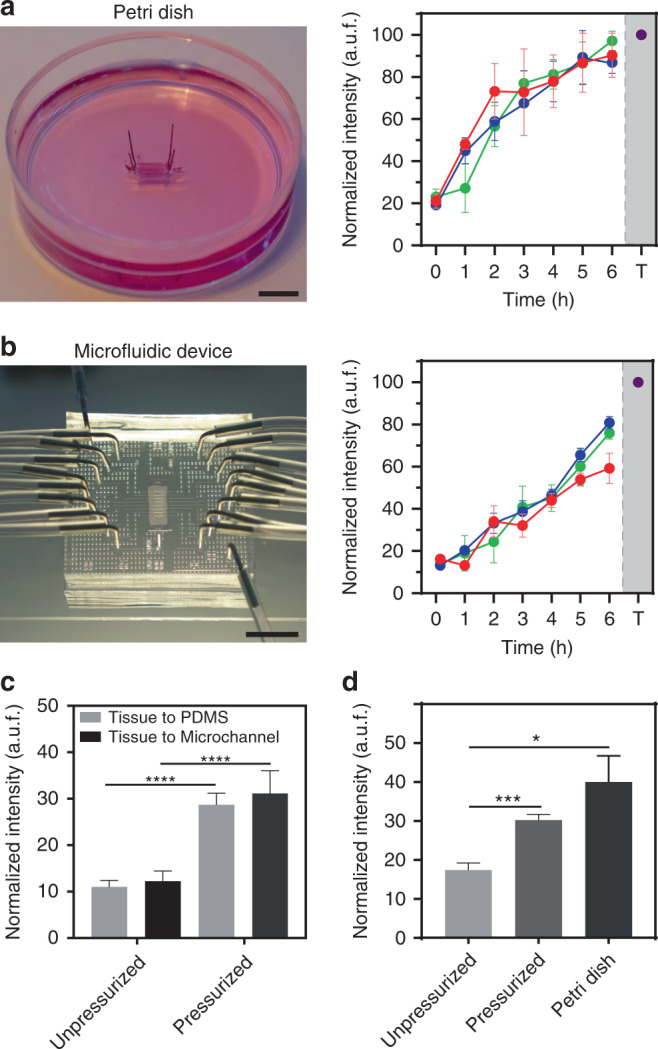
Comparison of tissue viability in a Petri dish vs microfluidic device. Tissue cell death was analyzed using EthD fluorescence intensity; Tween 20 (denoted as T) was used as a positive cell death control. Graphs show the results of aortic tissue cultured for 6 h in (**a**) a conventional static petri dish and (**b**) in our microfluidic device with constant perfusion. Each colored line represents a different tissue section. Error bars represent at least three measurements taken in different sections of the tissue. **c** Comparison of tissue viability in the microchannels and the PDMS interfaces before and after pressurization in the microfluidic device. **d** Comparison of tissue viability for 1 h under different conditions: in a device with the valve resting (unpressurized), with the valve actuated (pressurized) and in a Petri dish. *Indicates a *p*-value <0.05, and **** indicates a *p*-value < 0.0001. Scale bar: 5 mm

We assessed whether a tissue section could be cultured in the microfluidic device and compared its performance to conventional static culture techniques (culture dishes)^7^. Tissue death was evaluated using ethidium homodimer (EthD), a membrane impermeable DNA fluorescent dye that identifies cell death and damage. As a positive control, the tissue was exposed to 0.05% Tween 20 to permeabilize the cell membrane and induce cell death. An aorta section was anchored with needles on a petri dish covered with a thick layer of PDMS (Fig. 5a). The needles restricted tissue movement during media exchange and helped extend the tissue section 1 mm above the PDMS surface, so it was perfused with media on both sides. At the same time, we cultivated a tissue section (an aorta) in the device with continuous periperfusion for 7 h (37 °C with 5% CO_2_) without valve pressurization (Fig. 5b). The cell damage caused by tissue dissection and manipulation was assessed at the beginning of each experiment (*t* = 0). Remarkably, in our microfluidic device, tissue sections showed low levels of cell death signal for the first hour compared with tissue sections in the Petri dish, which showed a sharp increase in the cell death signal. Constant perfusion of the tissue with nutrients and washing away waste products promotes cell viability, while oxygen diffusion through the gas-permeable PDMS keeps the media oxygenated^7^. However, after the first hour, areas of the tissue sections rapidly started to die. Long-term cultures (several days), as seen in cannulated tissues^15^, could not be achieved with our device or with the Petri dish. Although the constant perfusion of nutrients can diffuse through a 200-µm-thick tissue section and keep the tissue section alive^7^, the thin PDMS layer located below the tissue inhibits media perfusion to the bottom surface. It is possible to replace this layer with a porous membrane to deliver nutrients and increase lifespan, as already noted in other reports^13,19^.

Next, we performed a viability assay with the valve actuated at 16 kPa since this is the pressure that aorta tissues experience under normal physiological conditions^26^, although the valve can be actuated at higher pressures to evaluate the effects of mechanical compression on ex vivo tissues that can lead to pathological effects. With an aorta inside the device, the valve was actuated at ~16 kPa for 1 h while media was continuously perfused. Then, the valve was deactivated, and EthD was flowed for 10 min before image acquisition (Fig. S7). We observed a twofold increase in the signal intensity of cell death under ~16 kPa compared with the control conditions (before valve pressurization, Fig. 5c). This significant increase in cell death was not observed in unpressurized tissue after 1 h (Fig. 5b). Remarkably, the areas outside the microchannels —where the tissue is in direct contact with PDMS— presented similar levels of tissue damage as areas of tissue that were constantly perfused through the microchannels. This effect could be attributed to cell–cell junctions or to transmural interstitial flow^30^. Furthermore, we found that compared with no compression in an unpressurized device, 1 h of tissue compression resulted in significant damage but significantly less damage than the conventional method (Fig. 5d). We hypothesize that the increase in tissue damage in our device could be caused mainly by the sudden compression of the tissue during prolonged exposure to a static high-pressure valve. Although the cell death levels were normalized and are useful to compare across different platforms, one must be careful interpreting these data. Tissue thickness irregularities do not allow us to focus evenly across the tissue section and prevent us from obtaining quantitative values of cell death. Overall, our valve-mediated device showed less cell damage than a static conventional method.

#### Drug dose–response assay

**Fig. 6:**
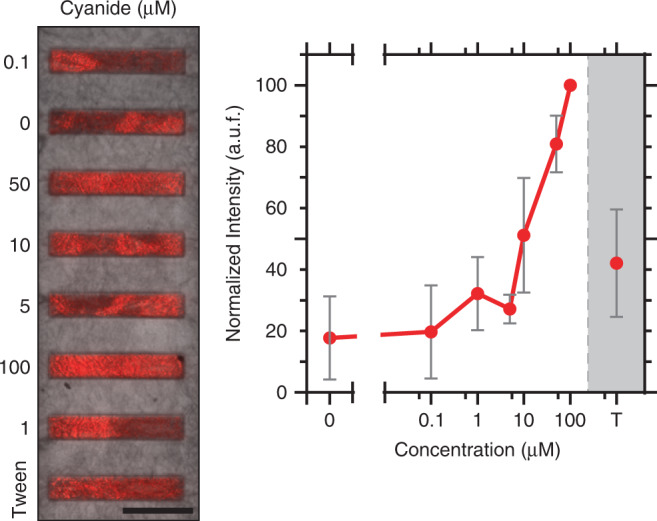
Dose–response assay validation using KCN poisoning. Merged brightfield and fluorescence micrographs of aortic tissue sections exposed for 30 min to seven different concentrations of KCN in parallel. Tween 20 (T) was used as a positive cell death control. Scale bar: 500 µm. Tissue damage was assessed with an ethidium homodimer. The graph shows the mean and standard deviation of four different aortic tissues

Toxicological assays study the action mechanism and effects associated with the exposure of biological samples to chemical agents. Particularly, in ex vivo assays, a sample is exposed to increasing concentrations (0.01 µM–1 mM) of a chemical agent for short (min-h) or long (days) periods, depending on the drug action mechanism, cell signaling pathway or cellular metabolism^31–33^. However, conventional toxicological assays require individual samples for each condition or concentration tested, limiting their applicability^7,13^. We tested the functionality of our device to deliver multiple concentrations of a drug to a single tissue sample. As a proof of concept, we used potassium cyanide (KCN), a drug that inhibits the production of adenosine triphosphate (ATP) in the mitochondrial respiratory chain, inducing cell apoptosis^34,35^. The tissue sections were exposed to six different concentrations of KCN (0, 0.1, 1, 5, 10, 50, and 100 µM) or 0.05% Tween 20 as a positive control at flow rates of 2.5 µL/min for 30 min with the valve actuated at ~16 kPa. To exclude contamination effects from contiguous channels, the concentrations were injected randomly in each of the 8 assay microchannels. Apoptotic and damaged cells were identified using EthD. As can be observed in Fig. 6, the fluorescence intensity was not homogeneous within the same assay microchannel, which can be attributed to some tissue regions being out of focus due to thickness irregularities. To compensate for these differences, the data were normalized to the channel with the highest fluorescence signal, assuming that EthD penetrates evenly throughout the sample. As expected, the fluorescence intensity increased proportionally to KCN dose; however, for low KCN concentrations (0.1 and 1 µM), it was difficult to discriminate between tissue damage caused by manual manipulation (0 µM) and drug effects (Figs. 6, S8). Although we expected similar levels of tissue damage between Tween 20 and the highest concentration of KCN, the response difference between these two molecules can be attributed to the cellular mechanisms they attack. KCN is a small molecule (65 Da) that diffuses rapidly into cells, blocking the mitochondrial respiratory chain, leading to an increase in radical oxygen species that damage the cell membrane much faster than Tween 20 and thus more effectively destroys cells^35^. In contrast, Tween 20 (1.2 kDa) interacts poorly with membrane rafts called detergent-resistant membranes (DRMs), which slows cell membrane permeabilization^36^. In summary, this experiment indicated that different drug concentrations can be tested simultaneously in a single ex vivo tissue section, albeit for short periods of time (min-h).

#### Proinflammatory response of the tunica intima

**Fig. 7:**
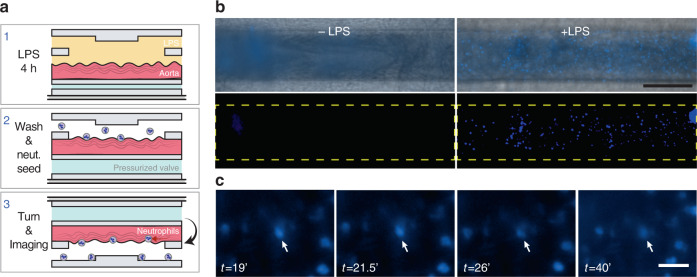
Proinflammatory induction of an aorta. **a** Experimental setup. (1) The aorta was exposed to LPS for 4 h. (2) After washing away LPS, the valve was actuated, and neutrophils (Hoechst stained) were perfused through the microchannels. (3) The device was flipped to allow unattached neutrophils to fall to the bottom surface and to later be removed. **b** Brightfield (top) and fluorescence (bottom) micrographs of the intima after exposure to buffer (left) or LPS (right). After 4 h, under control conditions, no neutrophils adhered to the intima, while several neutrophils adhered to the endothelia after LPS exposure. Scale bar: 250 µm. **c** Top view micrographs of the intima. The arrows point to a single neutrophil extravasating the tunica intima at different time points (min), revealed by its displacement along the *Z*-axis. Scale bar: 50 µm

Ex vivo studies open possibilities to mimic tissue inflammation and tumor metastatic events, allowing for the real-time observation of migratory cells (e.g., leukocytes, tumor cells, or pathogens) interacting with the tissue sample under different conditions^37,38^. As a complete organ-response experiment, we investigated the proinflammatory response of the endothelium when exposed to *E*. *coli* LPS (Fig. 7a). Briefly, the endothelium initiates a proinflammatory response when it detects LPS through the TLR4, CD14, and MD2 complex^39^. After 4 h, E-selectin, ICAM, and VCAM are expressed on the membrane of endothelial cells, enabling the recruitment of neutrophils, which extravasate the endothelium to reach the area of inflammation.

Two murine aorta sections were incubated in our device with either cell culture media or LPS for 4 h, while human neutrophils were isolated, purified, and stained with Hoechst. Next, neutrophils flowed through the channels of the device. After 3 min, the device was turned over to observe whether neutrophils had adhered to the tunica intima. In the LPS-stimulated tissue, we counted 272 activated neutrophils (Fig. 7b), some of which extravasated into the endothelium, while other cells moved across the tissue surface for over 30 min (Fig. 7c and Movie S1). After 40 min, 265 activated neutrophils were still adhered to the tissue surface of one of the microchannels (Fig. S9). Reproducibility was assessed on two more aortas from different mice on different days, identifying 48 and 13 neutrophils after 40 min of incubation (Figs. S10 and S11). Importantly, we did not observe the adhesion of neutrophils under control conditions (no LPS) in the three experiments performed. In summary, our microfluidic device can be used to study cell–tissue interactions and could be employed to investigate the intra- and extravasation mechanisms of inflammatory cells and circulating tumor cells.

### In vitro experimentation

#### Fluorescence immunohistochemical staining in a fixed tissue

Fluorescence immunohistochemistry (IHC) staining has been a critical tool in biomedicine and in the clinic for the identification and diagnosis of diseases^40^. This technique identifies proteins or antigens by either using labeled antibodies with high specificity (direct method) or primary antibodies followed by labeled secondary antibodies (indirect method). A crucial step in IHC is the preservation of the tissue with a fixative agent such as formaldehyde. Formaldehyde preserves tissues but also enables detergent permeabilization without cellular degradation, allowing antibodies to reach their specific intracellular protein target. We adapted an IHC staining protocol in our microfluidic device to verify the device’s ability to perform multiple staining assays in a single sample tissue.

**Fig. 8:**
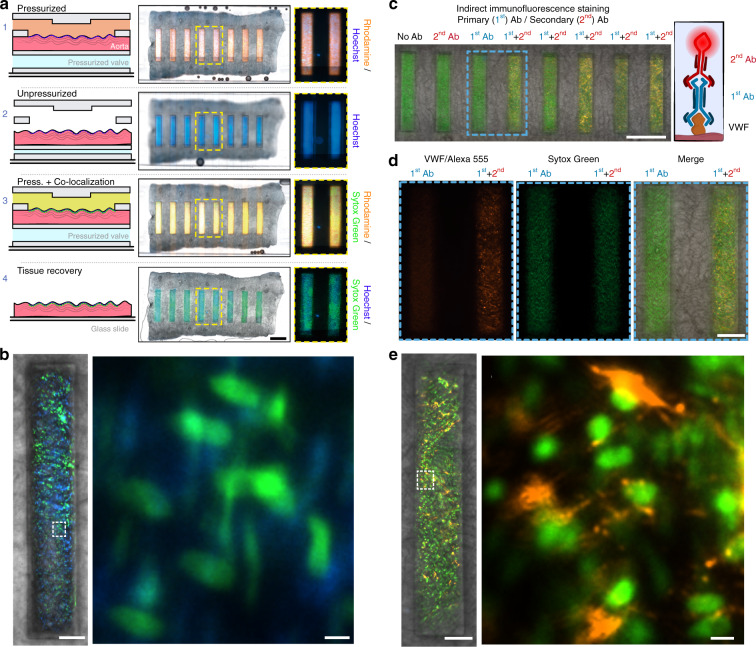
Parallel fluorescence immunohistochemical (IHC) staining inside our microfluidic platform. A fresh aortic tissue en face was fixed and permeabilized on the chip. **a** (1) Dextran-R and Hoechst were perfused through the 8 microchannels (step 1). (2) After washing away Dextran-R and Hoechst, the valve was depressurized, allowing for the detection of Hoechst-stained cells. (3) After valve repressurization, Dextran-R and Sytox Green overlapped with the Hoechst signal. (4) Tissue sections can be recovered from the microfluidic device for further analysis. Scale bar: 500 µm. **b** The nuclei of elongated smooth muscle stained with Hoechst (blue) are observably different from the rounded endothelial nuclei stained with Sytox (green). Right scale bar: 100 µm, left scale bar: 10 µm. **c** IHC for Von Willebrand factor on a fixed aorta en face. Cell nuclei were stained with Sytox Green; scale bar: 500 µm. **d** Magnification of the blue square in panel (**b**), highlighting some of the combinations tested. **e** Magnification of the indirect IHC staining of cell nuclei (Sytox Green) and Von Willebrand Factor IHC (Alexa Fluor 594, orange). Note that some cells are out of focus because of the surface irregularities of the tissue. Right scale bar: 100 µm, left scale bar: 10 µm

Fresh tissue (an aorta) was placed in the device and subjected to the following steps: fixation, permeabilization, blocking, staining and washing. The valve was kept actuated at ~34 kPa during the experiment. There was neither leakage nor diffusion of dyes (verified with Dextran-R and Hoechst, respectively) outside the assay microchannels after 10 min of perfusion (Fig. 8a-1), even though the tissue was fixed and permeabilized on-chip. To confirm that multiple staining steps can indeed be performed in the same location, we washed all the microchannels with PBS, and the valve was deactivated for 2 min (Fig. 8a-2). Next, the valve was actuated, and Sytox Green was flowed through all the channels (Fig. 8a-3). Notably, the fluorescence signals from all the channels colocalized to the fluorescence signal measured in the previous step, confirming that the tissue remains in the same position and thus allowing for multiple staining steps in the same channel (Fig. 8b). Finally, the tissue section can be recovered for downstream high-resolution imaging or molecular analysis by easily peeling off the top and bottom PDMS layers (Fig. 8a-4).

Next, we conducted an indirect IHC staining assay for Von Willerbrand factor (VWF) on a fresh fixed aorta in our device. VWF is a protein that plays a role in blood coagulation and wound healing and can be used as a biomarker to identify functional endothelial cells^41^. We conducted four different IHC assays on a single aortic tissue: (i) a control with no antibodies, (ii) incubation with fluorescently labeled secondary antibodies, (iii) incubation with primary antibodies, and (iv) a combination of primary and secondary antibodies (Fig. 8c). In all cases, cell nuclei were stained with Sytox Green. As expected, after incubation for 1 h, only the fourth condition showed a fluorescence signal in the red channel (corresponding to the fluorescent-labeled antibody), while the other conditions only presented a fluorescence signal in the green channel (Sytox Green) (Fig. 8c, d). No fluorescence signal was observed between channels. Furthermore, subcellular structures such as the nuclei (Fig. 8b, e) and VWF storage granules (Fig. 8e) can be clearly distinguished. Our approach reduces the number of tissue samples needed to carry out different assays and decreases IHC incubation times by at least fourfold compared with conventional protocols^5,25,42^. In summary, our device can perform indirect IHC staining assays; although only demonstrated for VWF, other proteins could be individually addressed in each assay channel, such as ICAM and ZO1.

## Conclusion

Ex vivo conventional techniques rely on static environments that alter nutrient supply and create oxygen gradients, detrimental for tissue culturing and impeding parallel experimentation on a single tissue sample^2,11–14^. Microfluidic devices have enabled the implementation of multiple assays on tissue sections, typically using slices with smooth surfaces, that nevertheless do not prevent crosstalk between channels^13,19^. We were able to overcome this problem by incorporating a tunable valve underneath the tissue section that, when actuated, creates eight independent channels running in parallel through the surface of the tissue. This strategy allowed us to conduct parallel assays over a single tissue sample unrestrained from its surface topography, preventing reagent leakage and diffusion to contiguous channels while being periperfused.

As demonstrated here, ex vivo tissue sections can be cultured for short periods of time (1 h), enough time to perform relevant ex vivo and in vitro assays. Although in its current configuration, our device cannot be used for long-term culture periods (>1 day)^15,23,43^, this can be overcome by fitting a porous membrane in the tissue chamber to increase the delivery of nutrients to the adventitia (bottom side of the tissue). In addition, small microchannels could be patterned between each assay microchannel to increase the delivery of nutrients to the apical side of the tissue^12,13^. Small molecules might diffuse into adjacent microchannels during long incubation periods (>1 h), but this can be mitigated using the same strategy: patterning intercalating channels to limit diffusion into contiguous assay microchannels. Furthermore, these intercalated microchannels will decrease contact between the tissue surface and the PDMS, reducing cell compression and thus cell death.

For ex vivo experimentation, we carried out tissue viability assays that showed a lower cell death signal in the microfluidic device than on static conventional culture plates. As a proof of concept for toxicological assays, different concentrations of cyanide were delivered simultaneously to a tissue section without cross-contamination between the eight microchannels. Nevertheless, the device must be further validated by exposing a single tissue to different drugs in parallel. We also demonstrated proinflammatory experiments in which neutrophils interacted with a tissue section, which could be easily extended to other assays, such as pathogen–tissue interactions^44^. Furthermore, we also performed complete immunohistochemical staining assays, a typical in vitro assay. We believe our device can be used to solve other relevant biological questions, such as tissue remodeling and restoration after tissue injury or revascularization^45,46^, and in biophysical tests, such as the study of mechanical compression effects (hypertensive in vitro model)^43^.

There are a couple of strategies that can be implemented to image subcellular structures, currently hindered by the thickness of the PDMS layers. The first strategy is to decrease the thickness of the valve and flow layers to 1 and 3 mm, respectively, which then offers the possibility of using long-distance objectives (6–12 mm working distance)^13^. A second strategy is to disassemble the device, retrieve the tissue, and place it on a cover slip for downstream analysis using epifluorescence and confocal microscopes. Ultimately, the chosen strategy will depend on the type of assay implemented.

Tissue-cut slices are poised to become an important tool to test drug efficacy, safety, and toxicology in humans before clinical trials^2,9,10^. Moreover, they are expected to facilitate studies in the areas of physiology, electrophysiology, metabolism, tissue regeneration and scarring. Furthermore, ex vivo approaches such as organ slice cultures can bridge the gap between in vitro cell culture and in vivo mammalian models to advance our understanding of biology. This microfluidic device can also aid in diagnostics by reducing the volume of reagents needed to run different tests and by allowing multiple assays to be performed on a single sample. Developing tools to facilitate the interrogation of tissue sections, irrespective of their physical and mechanical characteristics, such as the one presented here, will be critical to further promote the widespread use of tissue slices.

## Materials and methods

### Design and fabrication of the microfluidic device

Master molds were fabricated using standard photolithography techniques. First, four silicon wafers (4″, test-grade, Desert Silicon, USA) were cleaned in oxygen plasma (Zepto, Diener Electronic GmbH). Next, the flow and valve master molds were created by spin-coating a 25-µm layer of negative photoresist (GM1060, Gerstelec Sarl, Switzerland), while the tissue chamber and the assay microchannel master molds were coated with negative photoresist (SU-8 3050, MicroChem, USA) at the thicknesses of 250 and 150 µm, respectively. The wafers were prebaked as recommended by the manufacturer. The designs of each layer were patterned with a Micro Pattern Generator (µPG 101, Heidelberg Instruments). After a postbake step, the wafers were developed with PGMEA (484431, Sigma-Aldrich), followed by a hard-bake step of 2 h at 135 °C.

The microfluidic device was fabricated using multilayer soft lithography (Fig. S1). All master molds, acetate sheets (24075, Office Depot, Mexico) and steel press plates (diam.: 12 cm, height: 2.5 cm) were cleaned with isopropanol (V000139, Sigma-Aldrich) and blow-dried with N_2_ to eliminate any residues of PDMS or dust. The device is comprised of a top layer assembly (consisting of a flow layer and an assay microchannel layer) and a bottom layer assembly (composed of a tissue chamber layer and a valve layer). For the top assembly, a 10:1 weight ratio of PDMS elastomer and curing agent (Sylgard 184, Dow Corning) was mixed and poured on each of the molds. After degassing for 10 min, excess PDMS was removed from the assay microchannel mold before being sandwiched with two acetate sheets and press plates to create a thick porous membrane. The flow layer was partially cured at 80 °C for 21 min and cast out, and holes were punched and aligned to the assay microchannel layer. The assembly was cured for 2 h at 80 °C. For the bottom assembly, a weight ratio of 5:1 and 20:1 of elastomer to curing agent ratio was used for the valve and the tissue chamber layer, respectively. PDMS was poured on the tissue chamber mold and on the valve layer mold to heights of 500 μm and 3 mm, respectively, and cured at 80 °C for 21 min. After aligning the partially cured PDMS valve onto the tissue chamber layer, they were baked for 2 h at 80 °C. Next, inlets and outlets were punched out. During tissue dissection, the microchannel assay and tissue chamber layers were treated for 1.50 min with oxygen plasma before manually placing the tissue. The device is turned over and mounted on a motorized inverted microscope (Axio Observer A1, Zeiss Microscopy) to image the tissue surface.

### Imaging and statistical analysis

Micrographs were analyzed using ImageJ (ver. 1.52, National Institutes of Health, USA) to obtain intensity values in all channels. GraphPad Prism (ver. 7; GraphPad Software) was used to statistically analyze the data obtained from micrographs.

### Tissue dissection and deposition

All experiments with mice were approved by the Internal Committee of the Animal Care Facility of Cinvestav. The descending thoracic aortas and the trachea were dissected from healthy adult C57/B16 or C57BL/6-Tg(CAG-EGFP)131Osb/LeySopJ mice after cervical dislocation. Connective tissue surrounding the organ was removed in cold DMEM/F12 (11330032, Gibco) with 2% Pen/strep (15140-122, Thermo Fisher), followed by a transversal cut using microscissors (501233, McPherson-Vannas Scissors, World Precision Instruments) to expose the inner surface wall en face.

The tips of flat forceps (503233, Dumont Forceps, World Precision Instruments) were submerged in a 10:1 PDMS base-to-curing agent ratio. The forceps were placed inside the oven for 1 h at 80 °C in a vertical position with the tips pointing up and cured. This thin layer of PDMS prevents tissue adhesion and protects the tissue from forceps surface irregularities. Afterward, the forceps were submerged in alcohol and rinsed with 1X PBS before usage. The tissue was carefully placed on top of the flat PDMS-covered tip with the help of fine forceps (500342, Dumont #5, World Precision Instruments). Tissues were held from their corners with fine forceps to minimize tissue damage. Next, most of the liquid from the tissue was carefully drained by capillarity through the tips of the fine forceps. Then, the tissue was manually placed in the tissue chamber before sealing the top and bottom layer of the device. Finally, media was flowed in the flow channel of the tissue chamber layer.

### Histology

Aortas and tracheas en face were pinned to the surface of a petri dish coated with PDMS (10:1 ratio) using two acupuncture needles (PMX-GP, DBC, Korea) and were incubated overnight at 4 °C in 10% formalin (HT5011, Sigma) for fixation. Afterward, the aortas were frozen using PELCO Cryo-Embedding Compound (27300, Ted Pella), and 24-µm-thick slices were cut using a cryostat (CM 1100-3, Leica). Phase-contrast and fluorescence images were acquired with a motorized inverted microscope (Axio Observer A1, Zeiss Microscopy).

### Precision-cut tissue slices

After cervical dislocation, the murine brain was excised and cut into 2 × 6 mm blocks in cold PBS. Then, the tissue blocks were embedded in agarose (A9539, Sigma-Aldrich) and solidified for 15 min at 4 °C. Using a vibratome (752-888, Campden Instruments LTD, UK), 100-μm-thick slices were cut from each tissue block in a cold bath of PBS. The tissue was gently collected and placed in the tissue chamber. The valve was pressurized at 3.4, 6.9, and 13.8 kPa, while dextran-rhodamine was flowed through the assay microchannels.

### Device characterization

To characterize valve functionality, the assembly of the tissue chamber and the valve layers were bonded to a glass slide. A small PDMS slab (5 × 5 mm) was plasma-bonded on top of the valve inlet, and a hole was punched, through which blue food dye was injected. The valve maximum operating pressure was characterized by increasing the pressure in 6.9 kPa increments until the valve detached from the PDMS. Increasing membrane elevations were recorded with a USB digital microscope (Microcapture Pro 5MP, Celestron) from a side view of the valve. To study the elevation of the valve inside the tissue chamber, a finer characterization was conducted in steps of 0.7 kPa using a thin PDMS cylinder placed on the center of the valve. Micrographs were taken after pressure actuation.

To characterize the crosstalk between microchannels and detect any leaks between microchannels, dextran-conjugated rhodamine (40 kDa, D1842, Thermo Fisher) and fluorescein-5-isothiocyanate (389 Da, F7250, Sigma-Aldrich) were alternated in the 8 assay microchannels. For intercellular diffusion, fluorescent nuclear stains such as DAPI (62247, Thermo Fisher), Hoechst (H3570, Thermo Fisher) or Sytox Green (S7020, Thermo Fisher) were used in all assay microchannels. The valve was pressurized at 34.5 kPa.

### Tissue viability assay

Two aortic tissue culturing methods were tested for tissue viability: (1) aorta en face, fixed with acupuncture needles on a Petri dish, and (2) aorta en face in our microfluidic device. Ethidium homodimer (EthD-1, L3224, Thermo Fisher) was used as an indicator of cell death. Tissue media consisted of DMEM/F12 with 2% pen/strep. After dissection in fresh tissue media, the tissue was directly exposed for 10 min to media with EthD-1 at 37 °C and 5% CO_2_ followed by micrograph acquisition. Next, the tissue was cultured in tissue media for 7 h. Every hour, the tissue was exposed to EthD-1, and micrographs were acquired. As a positive cell death control, the tissue was exposed to DMEM with 0.05% Tween 20 (P9416, Sigma-Aldrich) for 10 min.

To study whether valve actuation affected tissue viability, aortas were placed in a device, and the valve was pressurized at a physiological systolic pressure of a murine aorta of 16.2 kPa^47^ for 1 h at 37 °C and 5% CO_2_. Next, the valve was deactivated, and tissue media with EthD-1 was perfused into the tissue chamber for 10 min before imaging.

After analyzing micrographs, the data were normalized by subtracting the difference in EthD-1 intensity signals at *t* = 0ʹ (cell damage due to tissue dissection and manipulation) and after EthD-1 exposure (*t* = Δ) and by dividing the result by the highest intensity value per experiment.

### Drug dose–response assay

A tunica intima sample was exposed to different concentrations of potassium cyanide (KCN) (60178, Sigma-Aldrich) with the valve actuated at 16.20 kPa. Each assay microchannel was perfused with 0, 2, 10, 20, 40, 80, or 100 mM KCN in tissue media for 30 min at 37 °C and 5% CO_2_. The 8th assay microchannel was used as a positive control for cell death by flowing 0.05% Tween 20. Finally, all assay microchannels were incubated for 10 min with EthD-1 and Hoechst.

To quantitate cell damage, we measured the average EthD-1 signal for each channel at two time points. First, immediately after placing the tissue in the device, E_*t*=0_, which is important to consider, as there can be significant tissue damage due to tissue dissection and manipulation. Then, after 30 min of incubation with the stimuli, E_*t*=30ʹ_. Next, we subtracted E_*t*=30ʹ_ from E_*t*=0_ for each channel and normalized this value to the channel with the highest intensity, which corresponded to the highest concentration of KCN.

### Proinflammatory response of the aorta inside the microfluidic device

A dissected descending aorta was cut in half, and each piece was placed in separate microfluidic devices. Inside the devices, one aorta en face was perfused for 4 h with tissue media containing 10 µg/mL lipopolysaccharide (LPS) from *E*. *coli* (L2755, Sigma-Aldrich, USA), while the second aorta was perfused with media as a control. The experiments were performed for 4 h at 37 °C and 5% CO_2_. Human neutrophils were isolated and purified from healthy donors as described elsewhere^48^. Purified neutrophils were stained with Hoechst and washed three times with Hank’s balanced salt solution (HBSS, ICN1810454, MP Biomedicals). LPS was washed out from the tissue chamber using fresh tissue media. The valve was actuated at 16.2 kPa before perfusing neutrophils into the assay microchannels at a flow rate of 2.5 µL/min. After 5 min, the flow was stopped, and the device was turned upside-down for video recording (Camtasia, TechSmith Corp.). Micrographs were captured every 15 s. Neutrophil identification and quantification analysis was performed using ImageJ. Brightness and contrast from the fluorescence micrographs were adjusted until the Hoechst signal from label cells was visible and the background signal was removed. Each labeled cell was numbered and identified through the whole stack of images.

### Immunohistochemical staining

Aortic tissue was fixed inside a microfluidic device with the valve actuated (34.5 kPa) using 10% formalin for 24 h at 4 °C. The valve remained actuated for the rest of the experiments. Then, the tissue was washed with tris(hydroxymethyl)aminomethane (TBS) buffer through the 8 assay microchannels, permeabilized with 0.05% Tween 20 for 30 min, and blocked with casein blocking solution (37582, Thermo Fisher) supplemented with 2% bovine serum albumin (BSA, A9647, Sigma-Aldrich) for 10 min. Next, the 8 microchannels were incubated for 3 h with the primary antibody against von Willerbrand factor (ab6994, Abcam). After washing, the assay microchannels were further incubated with Alexa-labeled secondary antibody (A-11012, Thermo Fisher) and Sytox Green for 1 h. Staining controls for the primary and secondary antibodies were performed in the same tissue by omitting their respective antibodies.

## Supplementary information

Supplementary material

Neutrophil Migration

graphical abstract

Editorial summary
