# Supplementary material for: A versatile microfluidic device for multiple ex vivo/in vitro tissue assays unrestrained from tissue topography
Source: Microsyst Nanoeng. 2020 Jun 29;6:40. doi: 10.1038/s41378-020-0156-0 (PMC8433291; doi:10.1038/s41378-020-0156-0)
Supplement: Supplementary file 1 — Supplementary material [file 41378_2020_156_MOESM1_ESM.docx]

**A versatile microfluidic device for multiple *ex vivo/in vitro* tissue assays unrestrained from tissue topography**

Jose M. de Hoyos-Vega^a^, Alan M. Gonzalez-Suarez^a^,

and Jose L. Garcia-Cordero^a§^

^a^ Unidad Monterrey, Centro de Investigación y de Estudios Avanzados del IPN,

Parque PIIT, NL, CP 66628, MEXICO

^§^Corresponding author: [jlgarciac@cinvestav.mx](mailto:jlgarciac@cinvestav.mx)

**Supplementary Information**

**Table 1:** List of reported microfluidic devices for *ex vivo/in vitro* studies

| **Tissue** | **Sampling** | **Experiment** | **Sample**  **size** | **Open/close**  **configuration** | **Perfusion/static** | **In chip Assays** | **Multiplex** | **Device**  **Material*** | **Year Ref.** |
| --- | --- | --- | --- | --- | --- | --- | --- | --- | --- |
| Brain | N/S | *Ex vivo* | 600 µm (t) | Open | Static | -Electrophysiology | No | SU-8 resist | 2003^1^ |
| Liver | -Krumdieck slicer | *Ex vivo* | 300 µm (t)  1 mm (t) | Close | 1.0 mL/min | -Propidium iodide diffusion  -Viability  -Toxicological | No | -PC  -PEEK  -Steel needles  -Teflon screw | 2007^2^ |
| Brain | -Vibratome | *Ex vivo* | 700 µm (t) | Open | 2-3 ml/min | -Electrophysiology | No | -PDMS | 2007^3^ |
| Brain | -Vibratome | *Ex vivo* | 350 µm (t) | Open | Static | - Voltammetric | Yes | -Slice anchor  -PDMS | 2008^4^ |
| Brain | N/S | *Ex vivo* | 350 µm (t) | Open | Static | -Tissue culture  -Axonal extension  -Electrophysiology | No | -PDMS | 2009^5^ |
| Brain | -Tissue chopper | *Ex vivo*  *In vitro* | 350 µm (t) | Open | Static | -Toxicological  -Electrophysiology  -IHC | No | -PDMS  -Petri dish | 2010^6^ |
| Breast cancer | -Microtome | *In vitro* | 4 µm (t) | Close | Perfusion  N/S | -IHC | Yes | -Weight  -Glass  -PDMS | 2010^7^ |
| Brain | -Vibratome | *Ex vivo* | 500 µm (t) | Close | 2.5 ml/min | -Electrophysiology | No | -PDMS | 2010^8^ |
| Liver | -Vibratome | *Ex vivo* | 4mm (d) 100 µm (t) | Close | 10µl/min | -Tissue culture  -Toxicological | No | -PC membranes and clamps  - screws  -PDMS | 2010^9^ |
|  |  |  |  |  |  |  |  |  |  |
|  |  |  |  |  |  |  |  |  |  |
|  |  |  |  |  |  |  |  |  |  |
| Brain | -Vibratome | *Ex vivo* | 400 µm (t) | Open | 0.0180 m/s. | -Electrophysiology  -Diffusion | Yes | -Platinum wire  -Filter paper  -PDMS | 2011^10^ |
| Liver | -Scalpel | *Ex vivo* | 4 mm^3^ | Close | 2 μl/min | -Toxicological | No | -English threaded adapter  -Glass  -PDMS | 2011^11^ |
| Brain | -Vibratome | *Ex vivo* | 400 µm (t) | Open | 13.5 ml/min | -Electrophysiology | No | -Porous polyester wool  - Perspex plates  - Silicon | 2011^12^ |
| Breast cancer | -Microtome | *In vitro* | 4 µm (t) | Close | 600 µl/h | -IHC | Yes | -PDMS | 2011^13^ |
| Brain | -Vibratome | *In vivo* | 350 µm (t) | Open | Perfusion/  static | Hypoxia detection | No | -PC  -PDMS | 2012^14^ |
| Breast cancer | -Microtome | *In vitro* | 5 µm (t) | Close | 10 to 100 μl/s | -IHC | No | -Screws  -PMMA  -Glass  -PDMS | 2013^15^ |
| Skin and hair | -Needle puncher | *Ex vivo* | 5 mm (d) | Open | 30 µl/min | -Tissue culture | No | -Transwell  -PDMS | 2013^16^ |
| Liver | -Tissue chopper | *Ex vivo* | 2 mm (t) | Close | 100 µl/min | -Tissue culture | No | - PEG-DA  - PMMA  -Glass | 2013^17^ |
| Brain | -Vibratome | *Ex vivo* | 300 µm (t) | Open | Static | -Diffusion  -Viability  -Toxicological | Yes | -PTFE porous membranes  - PS 96 well plate  -PDMS | 2014^18^ |
| Breast cancer | N/S | *In vitro* | 4 μm (t) | N/S | Perfusion | -IHC | NO | N/S | 2015^19^ |
| Adipose tissue | -Needle puncher  -Scalpel | *Ex vivo* | 3 mm (d) | Close | Static | -Tissue culture | No | -PP caps  -PDMS | 2015^20^ |
| Retina | -Whole tissue | *Ex vivo* | N/S | Open | Static | -Cell migration | Yes | -Agar gel  -Glass cylinder  -PDMS | 2015^21^ |
| Testis | -Whole tissue | *Ex vivo* | 1-2 mm | Close | 0.05 μl/min | -Tissue culture | No | - PC porous membrane  -PP tube  -PDMS | 2016^22^ |
| Brain | -Tissue chopper | *Ex vivo* | 350 μm (t) | Open | -Static  -42 μl/h | -Viability  -Electrophysiology | No | -MEA on glass  -PDMS | 2016^23^ |
| Intestine | -Needle puncher | *Ex vivo* | N/S | Close | 4 µl/min | -Permeability | No | -Glass  -PDMS | 2016^24^ |
| Tumor mouse xenografts | -Vibratome  -Needle puncher | *Ex vivo* | 300 μm (h)  380 μm (d) | Close | Static | -Viability  -Toxicological | No | -PDMS | 2016^25^ |
| Breast | -Needle core biopsy | *In vitro* | N/S | Close | N/S | -Protein extraction | No | -ITO-coated glass | 2017^26^ |
| Ureter, pancreas, prostate, breast carcinoma, pancreas and lung adenocarcinoma (NSCLC), and Hodgkin lymphoma | -N/S | *In vitro* | 4-10 μm (t) | Close | Perfusion | -IHC | No | -Gasket  -PDMS | 2017^27^ |
| Liver and kidney | -Tru-Cut biopsy needle  -Scalpel | *Ex vivo* | 1 cm (l)  1 mm (d) | Close | 20 ml/min | -Enzymatic digestion | No | -PDMS | 2017^28^ |
| Lymph node | -Vibratome | *Ex vivo* | 300 µm (t) | Open | 0.15 ml/min | -Diffusion  -Viability  -Delivery experiments | Yes | -PDMS | 2017^29^ |
| Epididymal adipose tissue pads | -Needle puncher | *Ex vivo* | 2-3 mm (d) | Open | Static | -Conductivity | No | -PDMS | 2017^30^ |
| Tumor, adipose tissue, and lymph nodes | -Vibratome | *Ex vivo* | 1-2 mm (d)  300 µm (h) | Close | 2 μl/ min | -Co-culture tissue communication | No | -PC porous membrane  - PMMA  -Glass  -PDMS | 2019^31^ |
| Human glioblastoma multiforme xenograft | -Vibratome | *Ex vivo* | 250 μm (t) | Open | Static | -Dilution | Yes | - PLA  - Nylon, polyester, cotton, and silk threads  - Sterlitech glass fiber membrane filter  - Transwell inserts | 2019^32^ |

**N/S**: Not specified; **t**: thickness; **d**: diameter; **l**: length; **IHC**: Immunohistochemical; **PC**: Polycarbonate; **PEEK**: PolyEtherEther-Ketone; PMMA: Poly methyl methacrylate; **PEG-DA**: Poly(ethylene glycol)-diacrylate polymer; **PTFE**: Hydrophilic polytetrafluoroethylene; **PS**: Polystyrene; **PP**: Polypropylene; **MEA**: Multiple electrode arrays, **ITO**: indium tin oxide; **PLA**: Polylactic acid. *Glass slides used as substrate and tubing were not taken in cons


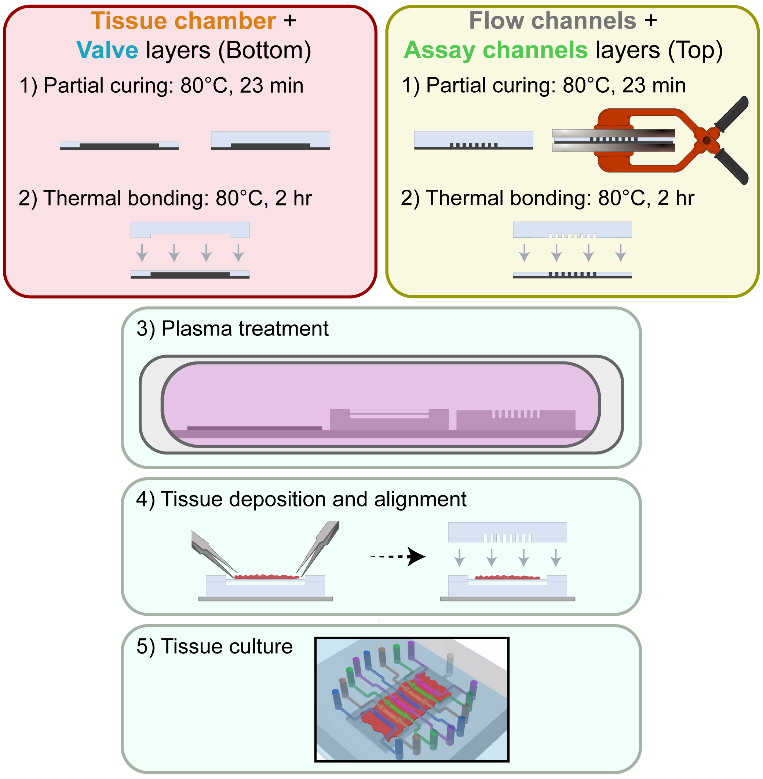


**Figure S1**: Step-by-step fabrication of the microfluidic device. **1**) PDMS is poured on all the master molds and partially cured. Assay microchannels are compressed between acetate sheets with a press to create two-sided open assay microchannels. **2**) The valve layer is casted out and aligned to the tissue chamber layer for thermal bonding. The flow layer is casted out and holes are punched before thermal bonding with the assay microchannel layer. **3**) Top and bottom layers are exposed to oxygen plasma after being separated from the mold. **4**) The tissue is gently deposited on the tissue chamber. Next, the top layer is aligned and bonded to the bottom layer, thus surrounding the tissue. **5**) Tissue chamber and flow channels are filled with culture media for further assay experimentation.


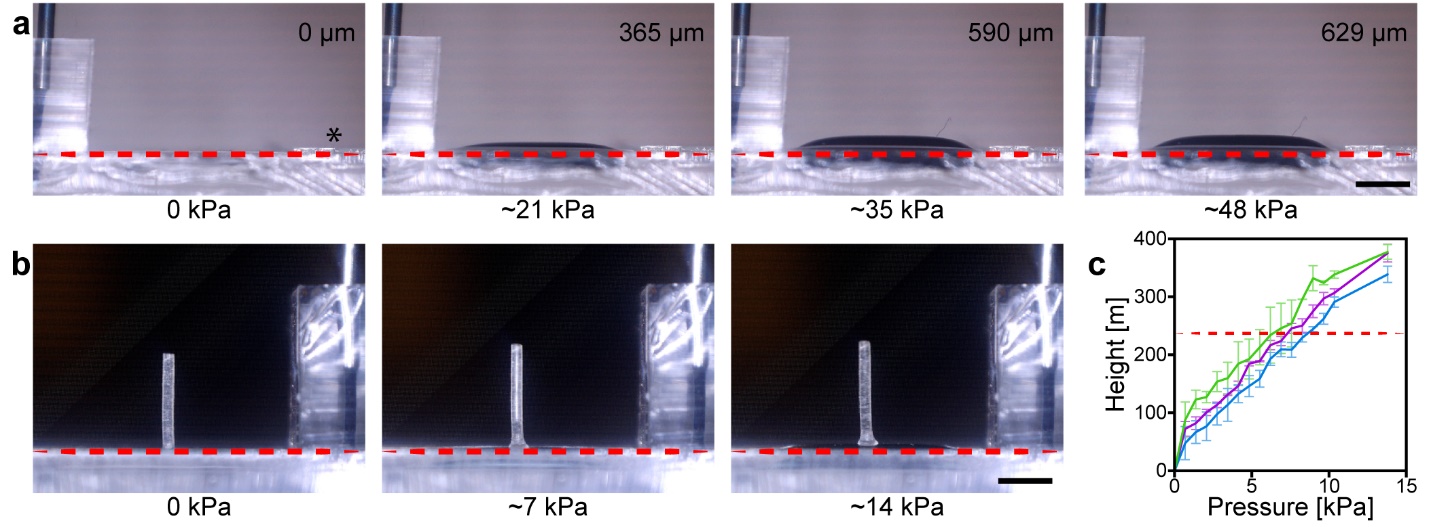


**Figure S2**: Characterization of heights reached by the valve at different pressures. (**a**) Micrograph series of the valve being actuated at different pressure values; scale bar chamber layer to detach from the valve layer.  (**b**) For low pressures and detect small movements, we placed a pin over the chamber. Micrographs show series of valve displacement inside the tissue chamber. (**c**) Plot of the valve displacement as a function of different applied pressures. Red horizontal line represents the tissue chamber depth.


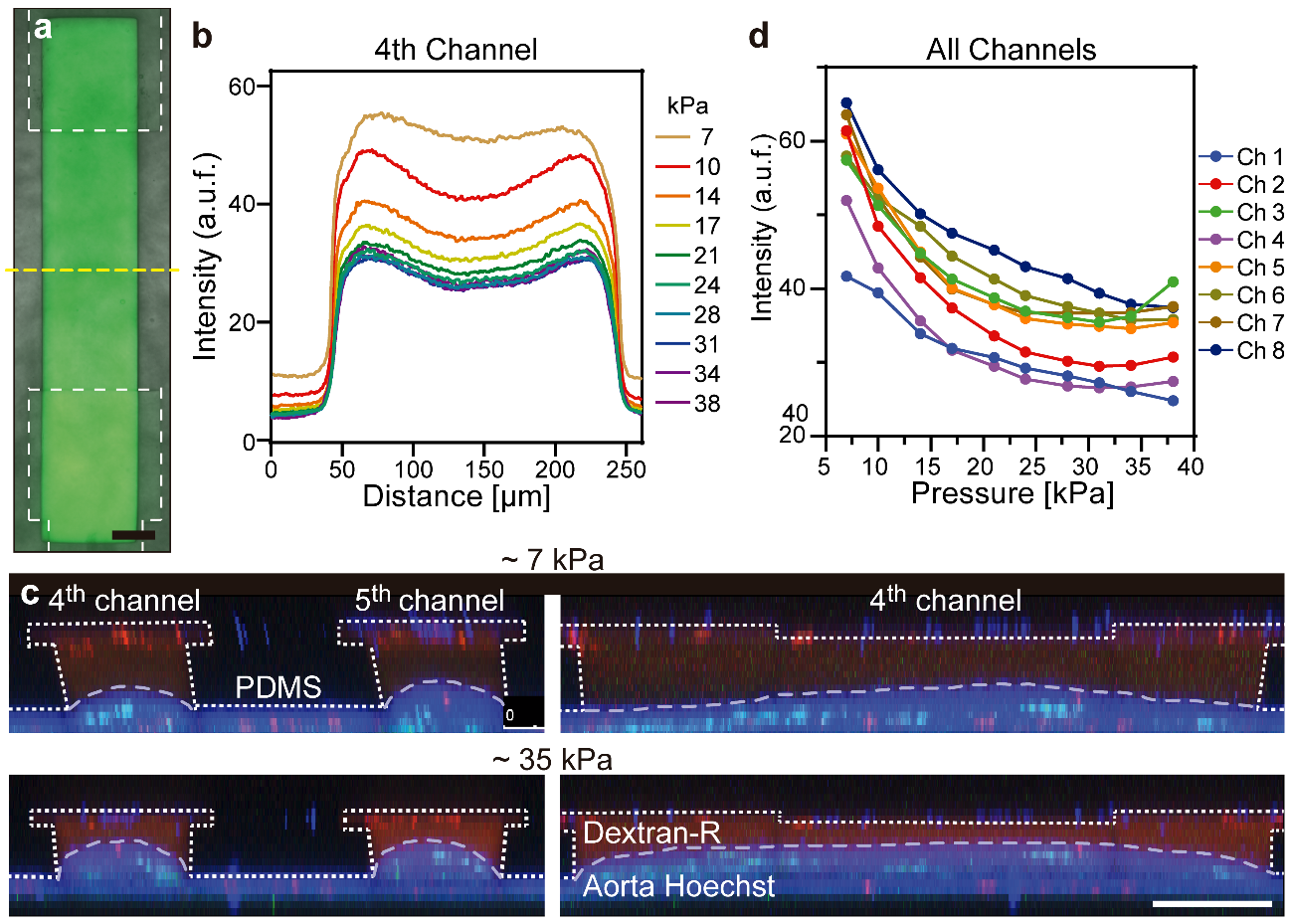


**Figure S3:**  Effects of valve deflection on tissue penetration into assay channels. (**a**) Micrograph of a typical assay channel being perfused with FITC with the valve actuated at ~7 kPa; scale bar: 100 µm. (**b**) Fluorescence intensity profiles measured at the center of the channel (dotted yellow line in image). Pressures on the valve are shown at the right of the graph. (**c**) Z-stack reconstruction of the transversal and longitudinal view of a microchannel (4th) with the aorta (Hoechst stained) being pressurized at ~7 and ~35 kPa. Microchannel was perfused with Dextran-R; scale bar: 250 µm.  (**d**) Fluorescence intensity values measured at the center of each microchannel at different valve pressures.


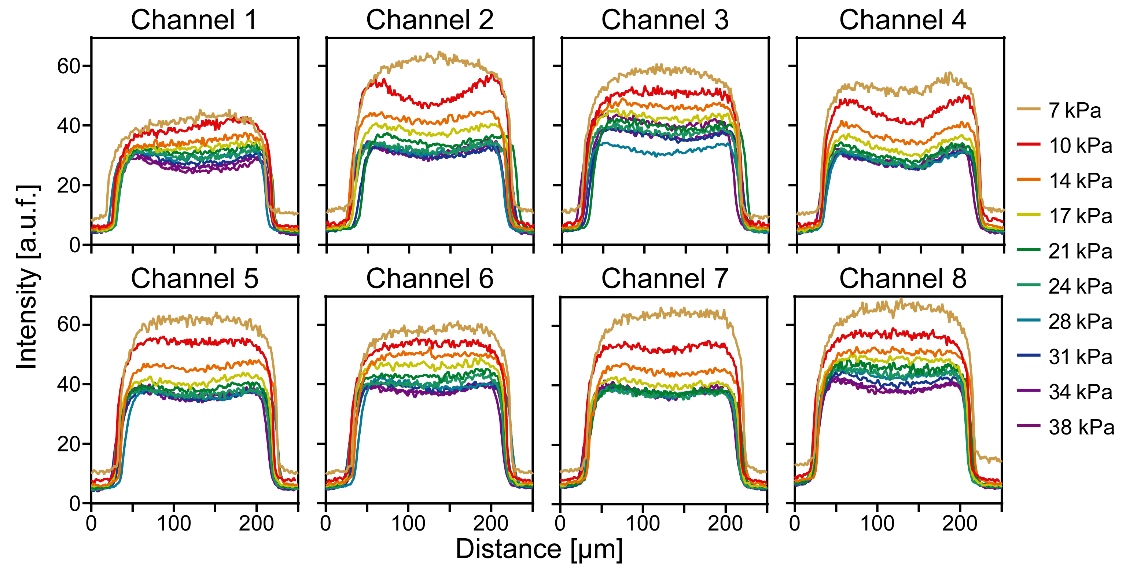


**Figure S4**: Effects of valve deflection on tissue penetration into the different assay channels of a single device. Fluorescence intensity profiles at the center of all 8 microchannels at different valve pressures ranging from ~7 kPa to ~35kPa. Leakage occurred below ~7 kPa.


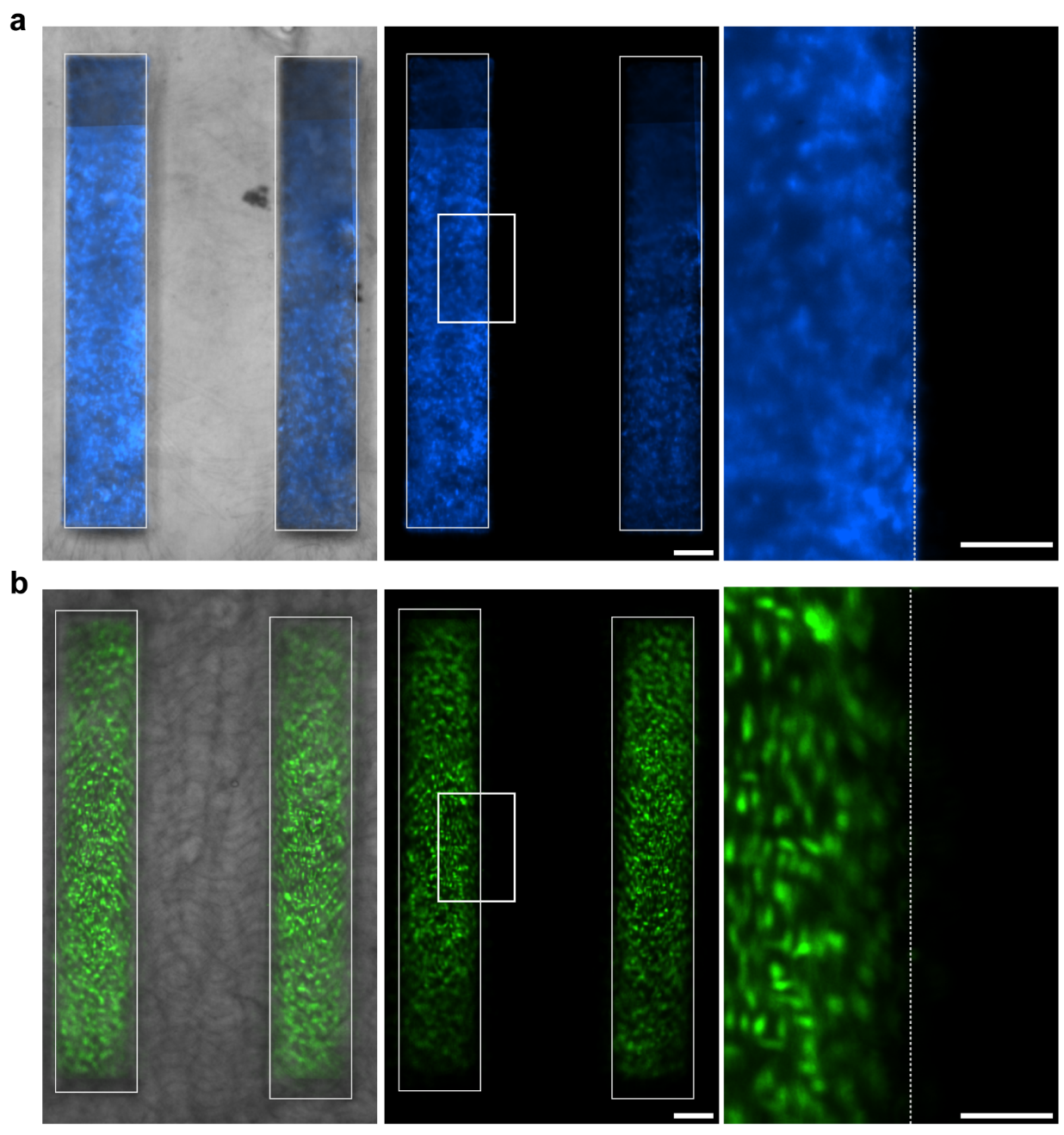


**Figure S5**: Magnification of microchannels assessing nucleic acid staining in (**a**) *ex vivo* tissue with Hoechst, and (**b**) in fixed aortic tissue with SYTOX green in the microfluidic device. Scale bars: left 100 µm and right 50 µm.


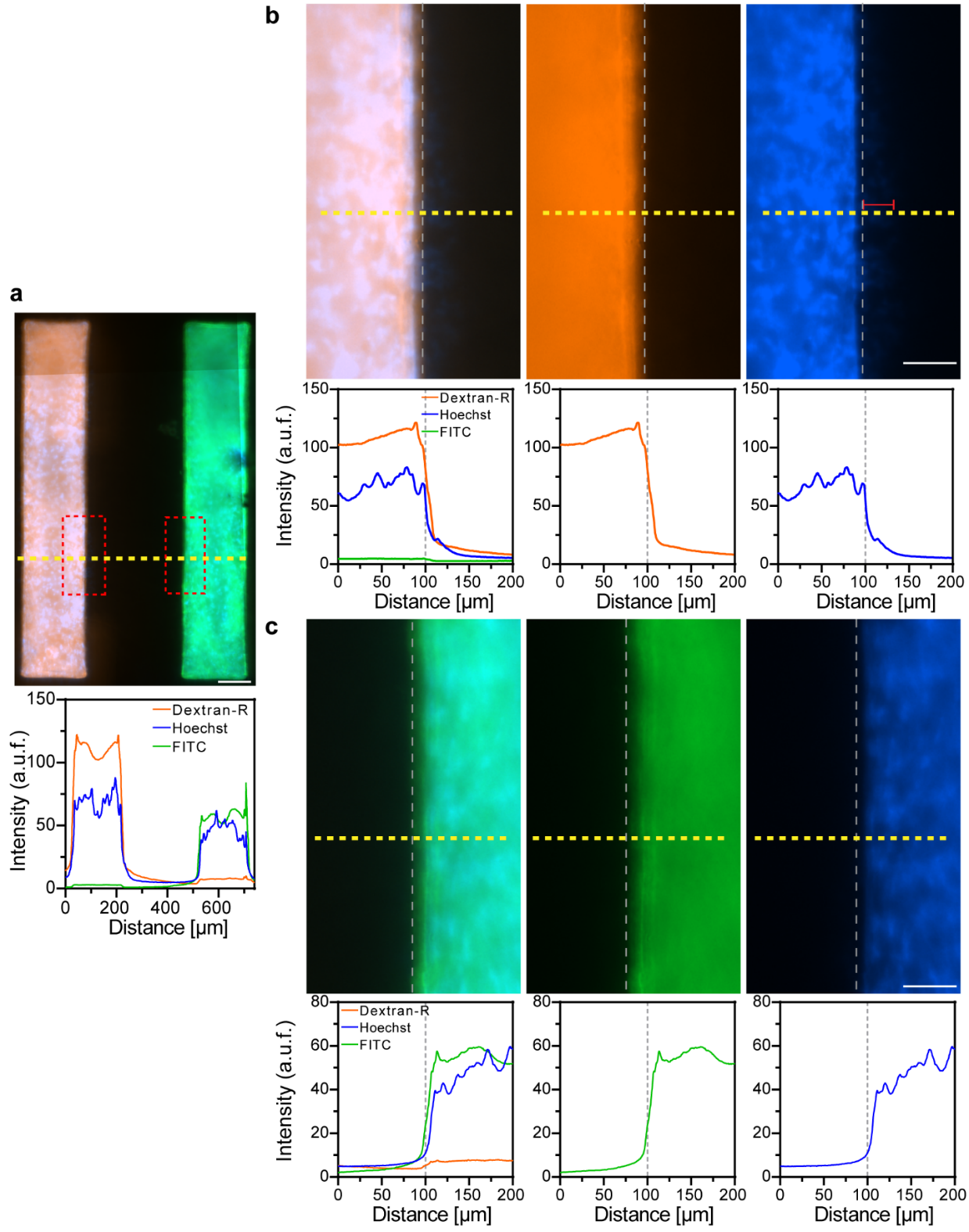


**Figure S6:** Leakage and diffusion assay on tissue section of an aorta with dye flow in the microfluidic device. (**a**) Brightfield and fluorescence micrographs of the tissue section in the chamber when Dextran-R and FITC in different channels. Hoechst dye was flowed in both channels. Scale bar: 100 µm. Fluorophores were perfused for 10 min at 2.85 µl/min. Fluorescence intensity profiles were measured across the horizontal dotted line. (**b**) Magnification of the left red square of micrograph (a). Vertical dotted lines define the microchannel’s wall; scale bar 50 µm. Dextran-R and Hoechst intensity profiles were measured across the horizontal dotted line. Hoechst lateral diffusion was traced up to 30 µm (red line on right micrograph), the dotted vertical line corresponds to microchannel’s wall. (**c**) Magnification of the right red square from fluorescence micrograph of (a); scale bar 50 µm. FITC and Hoechst intensity profiles were measured across the horizontal dotted line. The dotted vertical line corresponds to microchannel’s wall.


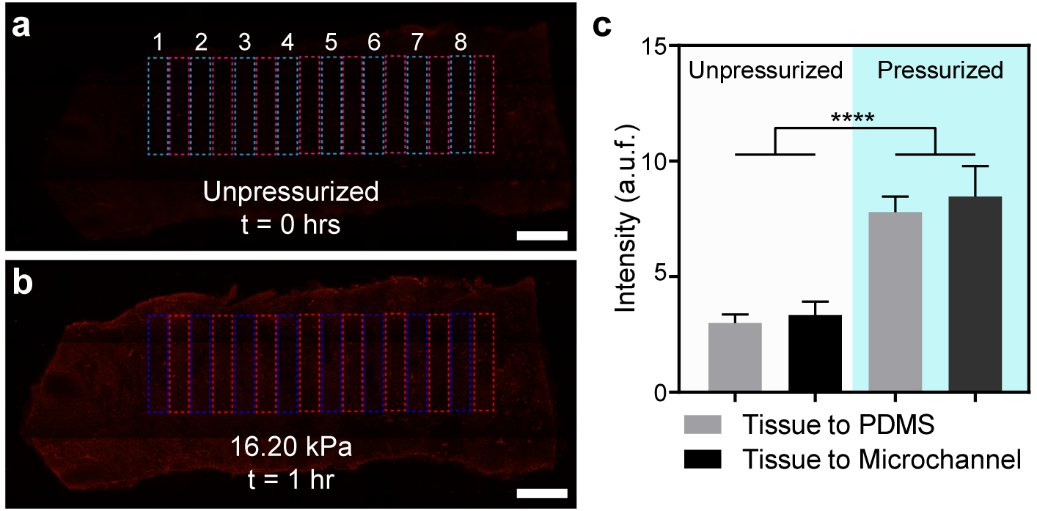


**Figure S7**: Tissue viability assay after valve pressurization.  (**a**) Fluorescent death staining (ethidium homodimer) on the aorta before valve pressurization; scale bar: 500 µm. Contact between tissue and PDMS is highlighted with blue dots, while the microchannels are highlighted with red dots. (**b**) Aorta was stained after 1 h of pressurization.


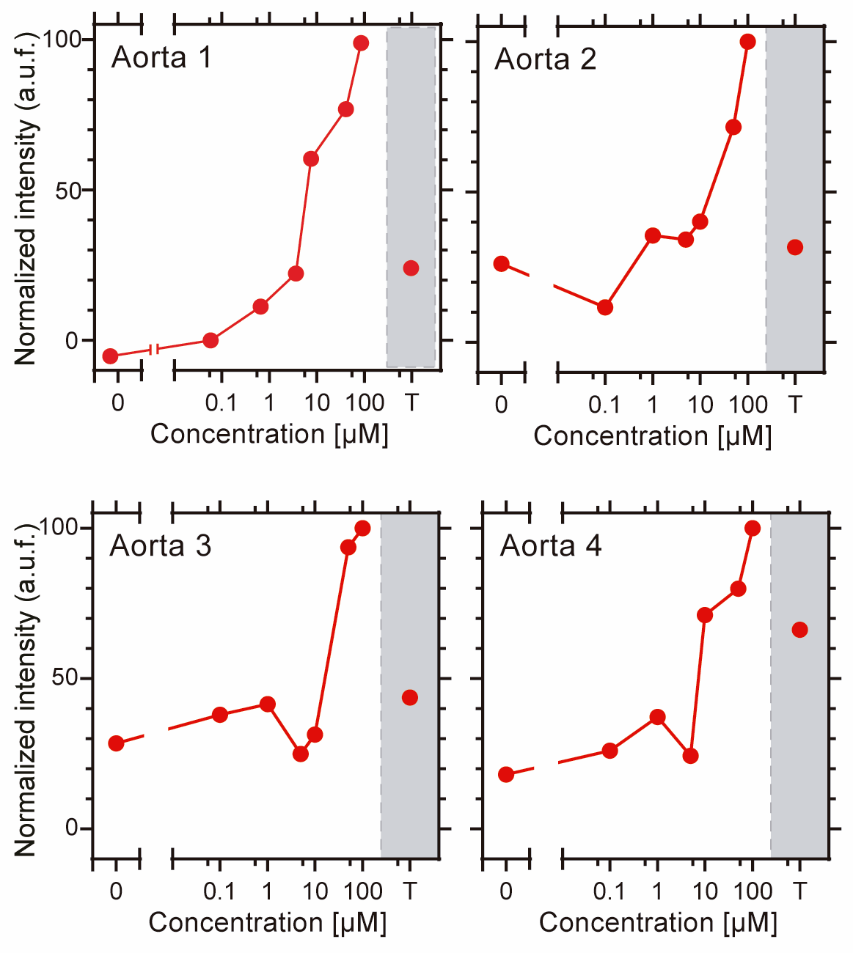


**Figure S8:** Results of a KCN dose-response assay for 4 different aortas. Aortic tissue was exposed to 7 different concentrations of KCN in parallel for 30 min. Tween 20 (T) was used as death control. Cell death was analyzed using ethidium homodimer.


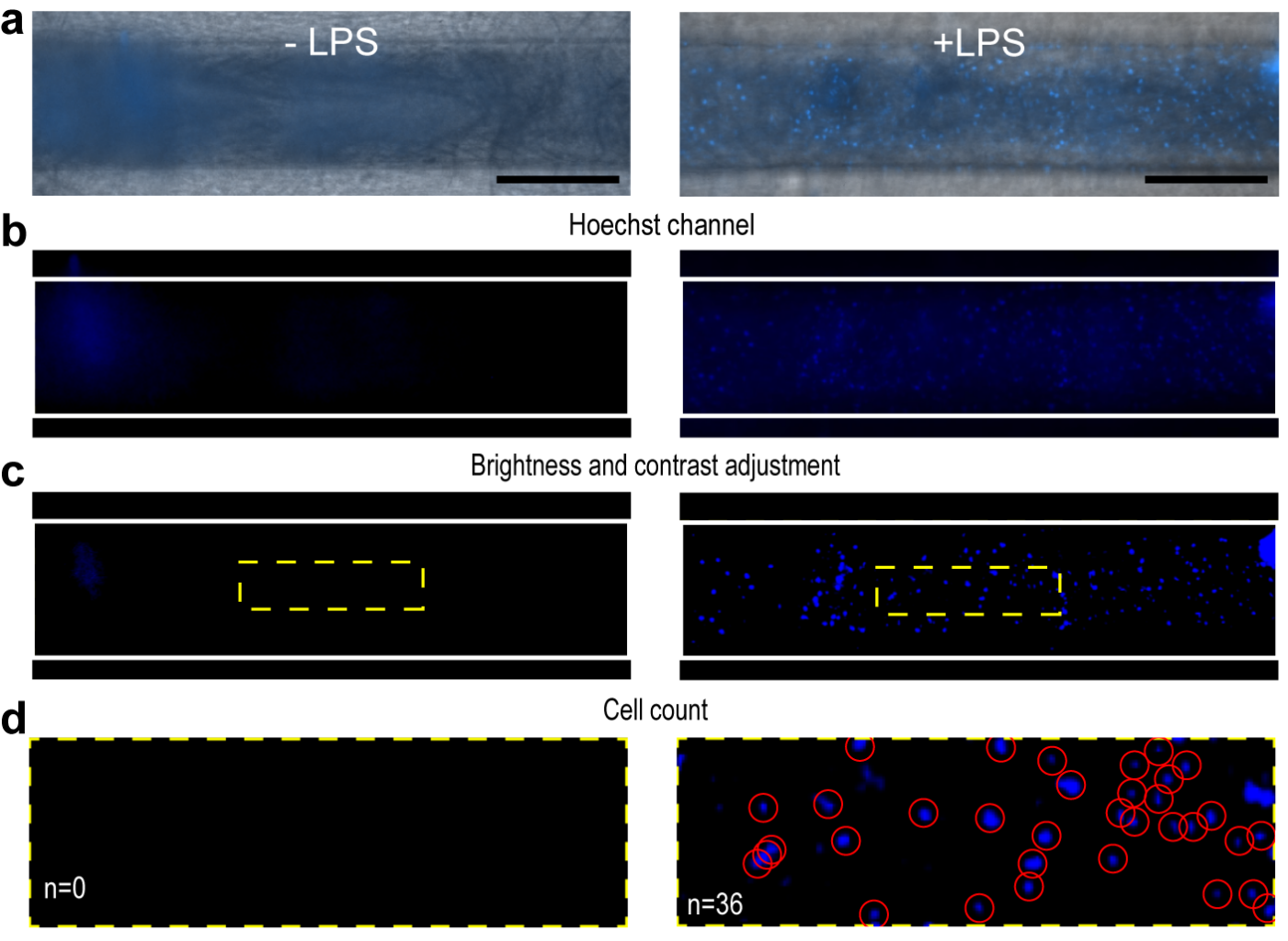


**Figure S9:** Supplementary data for Figure 7 showing adhesion of neutrophils to a tissue section in our microfluidic device. Hoechst-labeled neutrophils were flowed to the control (-LPS) and LPS-stimulated endothelium (+LPS). (**a**) Brightfield/fluorescence micrographs of the endothelium after 40 min of incubation. (**b**) Hoechst channel micrographs show the presence of neutrophils on the activated endothelium. Straight lines show the border of the channel. (**c**) Fluorescence brightness and contrast were adjusted to facilitate the identification of Hoechst-labeled neutrophils. (**d**) Zoom-in to regions denoted as a dotted rectangle in (c), showing neutrophils identified and circled in red. Scale bare: 250 µm.


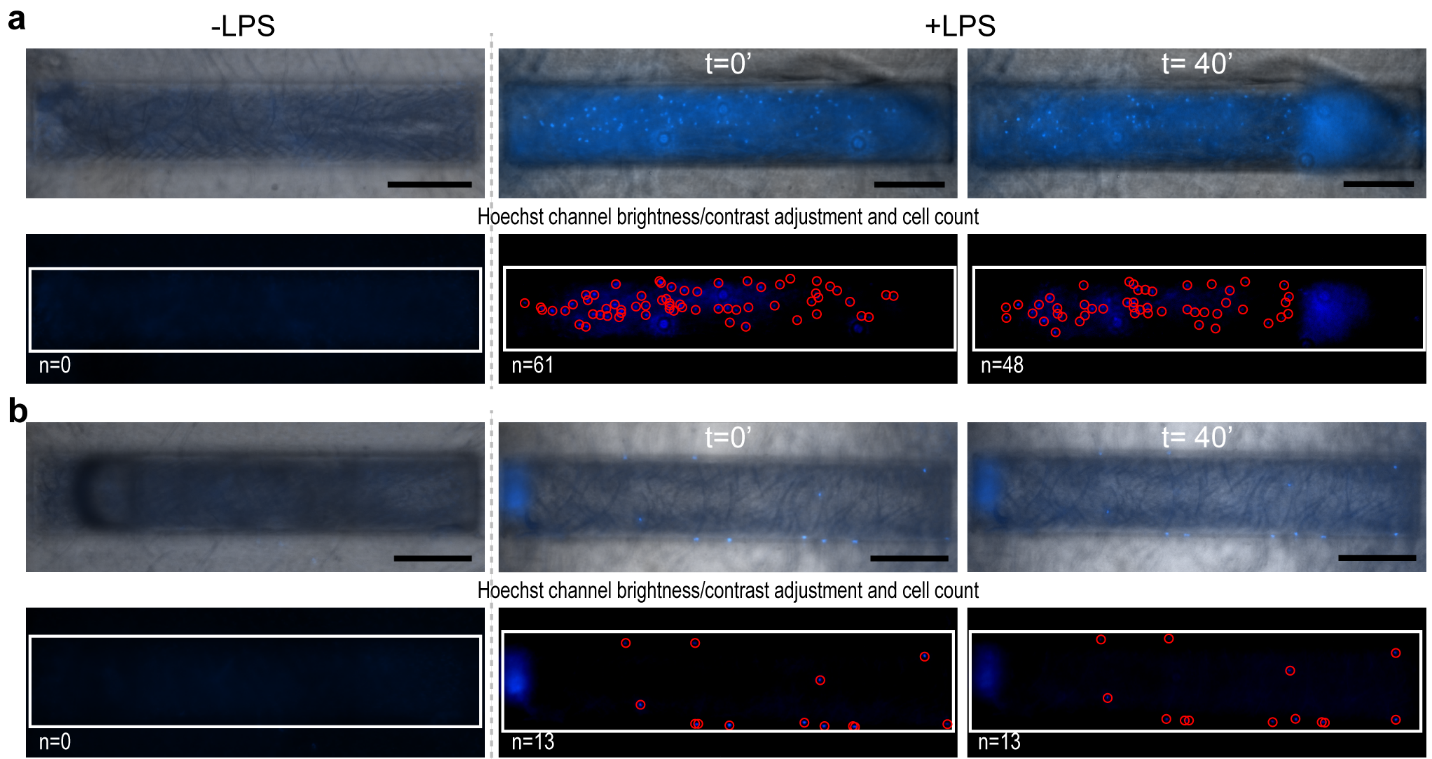


**Figure S10:** Reproducibility of the neutrophil adhesion assay on two murine aortas. Each assay of Hoechst-labeled neutrophils attached to LPS-stimulated endothelium (+LPS) were conducted in different days with their respective controls (-LPS). (**a**) No neutrophils were observed to attach on the control assay. Adhesion of 61 neutrophils were identified on stimulated tissue sections at the start of the assay. After 40 min of incubation, 48 neutrophils remained on the endothelium surface. Scale bare: 250 µm. (**b**) Adhesion of 13 neutrophils were identified after 40 min of incubation in an LPS-treated aorta. No neutrophils were observed to adhere on control conditions. Scale bare: 250 µm.


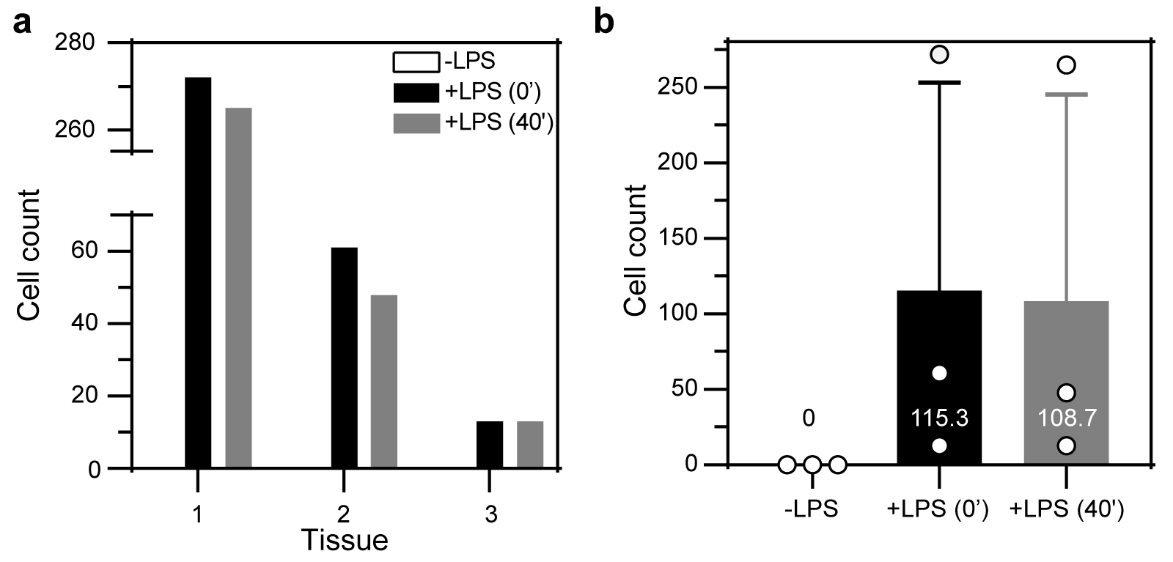


**Figure S11:** Comparison of the number of neutrophils attached to three aorta sections. Results of neutrophils adhered to tissue section stimulated with LPS or PBS. Neutrophils were counted after being introduced in the chip (0 min) or after 40 min.

**References**

(1) Passeraub, P.; Almeida, A.; Thakor, N. Design , Microfabrication and Analysis of a Microfluidic Chamber for the Perfusion of Brain Tissue Slices. *Biomed. Microdevices* **2003**, *5* (147), 147–155. https://doi.org/1024547413721.

(2) Khong, Y. M.; Zhang, J.; Zhou, S.; Cheung, C.; Doberstein, K.; Samper, V.; Yu, H. Novel Intra-Tissue Perfusion System for Culturing Thick Liver Tissue. *Tissue Eng.* **2007**, *13* (9), 2345–2356. https://doi.org/10.1089/ten.2007.0040.

(3) Blake, A. J.; Pearce, T. M.; Rao, N. S.; Johnson, S. M.; Williams, J. C. Multilayer PDMS Microfluidic Chamber for Controlling Brain Slice Microenvironment. *Lab Chip* **2007**, *7* (7), 842. https://doi.org/10.1039/b704754a.

(4) Mohammed, J. S.; Caicedo, H. H.; Fall, C. P.; Eddington, D. T. Microfluidic Add-on for Standard Electrophysiology Chambers. *Lab Chip* **2008**, *8* (7), 1048–1055. https://doi.org/10.1039/b802037j.

(5) Berdichevsky, Y.; Sabolek, H.; Levine, J. B.; Staley, K. J.; Yarmush, M. L. Microfluidics and Multielectrode Array-Compatible Organotypic Slice Culture Method. **2009**, *178*, 59–64. https://doi.org/10.1016/j.jneumeth.2008.11.016.

(6) Berdichevsky, Y.; Staley, K. J.; Yarmush, M. L. Building and Manipulating Neural Pathways with Microfluidics. *Lab Chip* **2010**, *10* (8), 999. https://doi.org/10.1039/b922365g.

(7) Kim, M. S.; Kim, T.; Kong, S.-Y.; Kwon, S.; Bae, C. Y.; Choi, J.; Kim, C. H.; Lee, E. S.; Park, J.-K. Breast Cancer Diagnosis Using a Microfluidic Multiplexed Immunohistochemistry Platform. *PLoS One* **2010**, *5* (5), e10441. https://doi.org/10.1371/journal.pone.0010441.

(8) Blake, A. J.; Rodgers, F. C.; Bassuener, A.; Hippensteel, J. A.; Pearce, T. M.; Pearce, T. R.; Zarnowska, E. D.; Pearce, R. A.; Williams, J. C. A Microfluidic Brain Slice Perfusion Chamber for Multisite Recording Using Penetrating Electrodes. *J. Neurosci. Methods* **2010**, *189* (1), 5–13. https://doi.org/10.1016/j.jneumeth.2010.02.017.

(9) Van Midwoud, P. M.; Groothuis, G. M. M.; Merema, M. T.; Verpoorte, E. Microfluidic Biochip for the Perifusion of Precision-Cut Rat Liver Slices for Metabolism and Toxicology Studies. *Biotechnol. Bioeng.* **2010**, *105* (1), 184–194. https://doi.org/10.1002/bit.22516.

(10) Tang, Y. T.; Kim, J.; López-Valdés, H. E.; Brennan, K. C.; Ju, Y. S. Development and Characterization of a Microfluidic Chamber Incorporating Fluid Ports with Active Suction for Localized Chemical Stimulation of Brain Slices. *Lab Chip* **2011**, *11* (13), 2247. https://doi.org/10.1039/c1lc20197b.

(11) Hattersley, S. M.; Greenman, J.; Haswell, S. J. Study of Ethanol Induced Toxicity in Liver Explants Using Microfluidic Devices. *Biomed Microdevices* **2011**, *13* (6), 1005–1014. https://doi.org/10.1007/s10544-011-9570-2.

(12) Hill, M. R. H.; Greenfield, S. A. The Membrane Chamber: A New Type of in Vitro Recording Chamber. *J. Neurosci. Methods* **2011**, *195* (1), 15–23. https://doi.org/10.1016/j.jneumeth.2010.10.024.

(13) Kim, M. S.; Kwon, S.; Kim, T.; Lee, E. S.; Park, J. K. Quantitative Proteomic Profiling of Breast Cancers Using a Multiplexed Microfluidic Platform for Immunohistochemistry and Immunocytochemistry. *Biomaterials* **2011**, *32* (5), 1396–1403. https://doi.org/10.1016/j.biomaterials.2010.10.040.

(14) Mauleon, G.; Fall, C. P.; Eddington, D. T. Precise Spatial and Temporal Control of Oxygen within In Vitro Brain Slices via Microfluidic Gas Channels. *PLoS One* **2012**, *7* (8), e43309. https://doi.org/10.1371/journal.pone.0043309.

(15) Ciftlik, A. T.; Lehr, H.-A.; Gijs, M. a M. Microfluidic Processor Allows Rapid HER2 Immunohistochemistry of Breast Carcinomas and Significantly Reduces Ambiguous (2+) Read-Outs. *Proc. Natl. Acad. Sci. U. S. A.* **2013**, *110*, 5363–5368. https://doi.org/10.1073/pnas.1211273110.

(16) Horland, R.; Wagner, I.; Lauster, R.; Tonevitsky, A. G.; Azar, R. P.; Ataç, B.; Marx, U.; Lindner, G. Skin and Hair On-a-Chip: In Vitro Skin Models versus Ex Vivo Tissue Maintenance with Dynamic Perfusion. *Lab Chip* **2013**, *13* (18), 3555. https://doi.org/10.1039/c3lc50227a.

(17) Sivashankar, S.; Puttaswamy, S. V.; Lin, L. H.; Dai, T. S.; Yeh, C. T.; Liu, C. H. Culturing of Transgenic Mice Liver Tissue Slices in Three-Dimensional Microfluidic Structures of PEG-DA (Poly(Ethylene Glycol) Diacrylate). *Sensors Actuators, B Chem.* **2013**, *176*, 1081–1089. https://doi.org/10.1016/j.snb.2012.09.087.

(18) Chang, T. C.; Mikheev, A. M.; Huynh, W.; Monnat, R. J.; Rostomily, R. C.; Folch, A. Parallel Microfluidic Chemosensitivity Testing on Individual Slice Cultures. *Lab Chip* **2014**, *14*, 4540–4551. https://doi.org/10.1039/C4LC00642A.

(19) Kwon, S.; Cho, C. H.; Lee, E. S.; Park, J. Automated Measurement of Multiple Cancer Biomarkers Using Quantum-Dot-Based Micro Fl Uidic Immunohistochemistry. **2015**. https://doi.org/10.1021/acs.analchem.5b00199.

(20) Zambon, A.; Zoso, A.; Gagliano, O.; Magrofuoco, E.; Fadini, G. P.; Avogaro, A.; Foletto, M.; Quake, S.; Elvassore, N. High Temporal Resolution Detection of Patient-Specific Glucose Uptake from Human Ex Vivo Adipose Tissue On-Chip. *Anal. Chem.* **2015**, *87* (13), 6535–6543. https://doi.org/10.1021/ac504730r.

(21) Dodson, K. H.; Echevarria, F. D.; Li, D.; Sappington, R. M.; Edd, J. F. Retina-on-a-Chip: A Microfluidic Platform for Point Access Signaling Studies. *Biomed. Microdevices* **2015**, *17* (6), 1–10. https://doi.org/10.1007/s10544-015-0019-x.

(22) Komeya, M.; Kimura, H.; Nakamura, H.; Yokonishi, T.; Sato, T.; Kojima, K.; Hayashi, K.; Katagiri, K.; Yamanaka, H.; Sanjo, H.; et al. Long-Term Ex Vivo Maintenance of Testis Tissues Producing Fertile Sperm in a Microfluidic Device. *Sci. Rep.* **2016**, *6* (February), 1–10. https://doi.org/10.1038/srep21472.

(23) Liu, J.; Pan, L.; Cheng, X.; Berdichevsky, Y. Perfused Drop Microfluidic Device for Brain Slice Culture-Based Drug Discovery. *Biomed. Microdevices* **2016**. https://doi.org/10.1007/s10544-016-0073-z.

(24) Dawson, A.; Dyer, C.; Macfie, J.; Davies, J.; Karsai, L.; Greenman, J.; Jacobsen, M. A Microfluidic Chip Based Model for the Study of Full Thickness Human Intestinal Tissue Using Dual Flow. *Biomicrofluidics* **2016**, *10* (6), 1–10. https://doi.org/10.1063/1.4964813.

(25) Astolfi, M.; Péant, B.; Lateef, M. A.; Rousset, N.; Kendall-Dupont, J.; Carmona, E.; Monet, F.; Saad, F.; Provencher, D.; Mes-Masson, A. M.; et al. Micro-Dissected Tumor Tissues on Chip: An Ex Vivo Method for Drug Testing and Personalized Therapy. *Lab Chip* **2016**, *16* (2), 312–325. https://doi.org/10.1039/c5lc01108f.

(26) Abdulwahab, S.; Ng, A. H. C.; Dean Chamberlain, M.; Ahmado, H.; Behan, L. A.; Gomaa, H.; Casper, R. F.; Wheeler, A. R. Towards a Personalized Approach to Aromatase Inhibitor Therapy: A Digital Microfluidic Platform for Rapid Analysis of Estradiol in Core-Needle-Biopsies. *Lab Chip* **2017**, *17* (9), 1594–1602. https://doi.org/10.1039/c7lc00170c.

(27) Brajkovic, S.; Dupouy, D. G.; de Leval, L.; Gijs, M. A. Microfluidics for Rapid Cytokeratin Immunohistochemical Staining in Frozen Sections. *Lab. Investig.* **2017**, *97* (8), 983–991. https://doi.org/10.1038/labinvest.2017.49.

(28) Qiu, X.; Westerhof, T. M.; Karunaratne, A. A.; Werner, E. M.; Pourfard, P. P.; Nelson, E. L.; Hui, E. E.; Haun, J. B. Microfluidic Device for Rapid Digestion of Tissues into Cellular Suspensions. *Lab Chip* **2017**, *17* (19), 3300–3309. https://doi.org/10.1039/C7LC00575J.

(29) Ross, A. E.; Belanger, M. C.; Woodroof, J. F.; Pompano, R. R. Spatially Resolved Microfluidic Stimulation of Lymphoid Tissue: Ex Vivo. *Analyst* **2017**, *142* (4), 649–659. https://doi.org/10.1039/c6an02042a.

(30) Li, X.; Brooks, J. C.; Hu, J.; Ford, K. I.; Easley, C. J. 3D-Templated, Fully Automated Microfluidic Input/Output Multiplexer for Endocrine Tissue Culture and Secretion Sampling. *Lab Chip* **2017**, *17* (2), 341–349. https://doi.org/10.1039/c6lc01201a.

(31) Harris, A. R.; Shim, S.; Pompano, R. R.; Munson, J.; Belanger, M. C. Two-Way Communication between Ex Vivo Tissues on a Microfluidic Chip: Application to Tumor-Lymph Node Interaction. *Lab Chip* **2019**. https://doi.org/10.1039/c8lc00957k.

(32) Rumaner, M.; Horowitz, L.; Ovadya, A.; Folch, A. Thread as a Low-Cost Material for Microfluidic Assays on Intact Tumor Slices. *Micromachines* **2019**, *10* (481). https://doi.org/10.3390/mi10070481.
