# Supplementary figures and images for: A versatile microfluidic device for multiple ex vivo/in vitro tissue assays unrestrained from tissue topography
Source: Microsyst Nanoeng. 2020 Jun 29;6:40. doi: 10.1038/s41378-020-0156-0 (PMC8433291; doi:10.1038/s41378-020-0156-0)

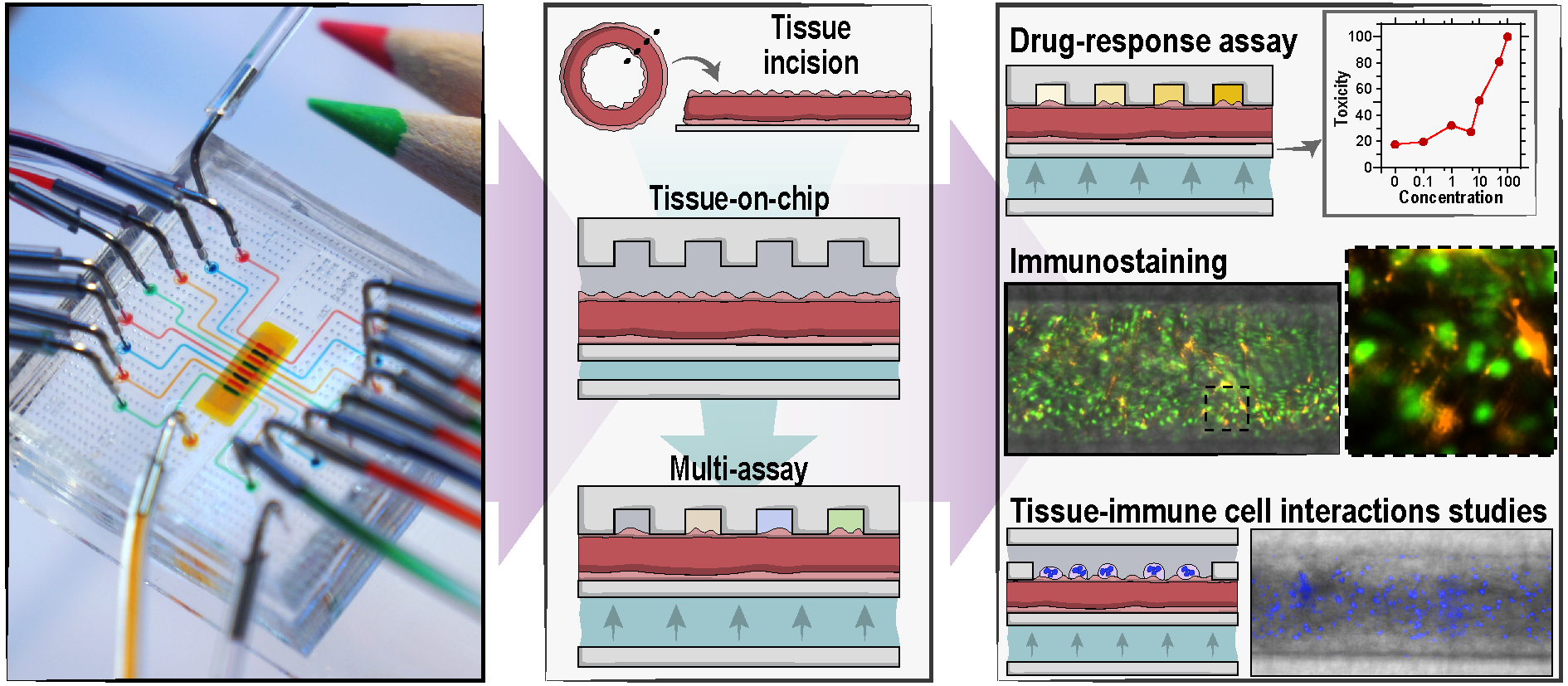

Supplement: Supplementary file 3 — graphical abstract [file 41378_2020_156_MOESM3_ESM.tif]
