# Supplementary material for: A versatile microfluidic device for multiple ex vivo/in vitro tissue assays unrestrained from tissue topography
Source: Microsyst Nanoeng. 2020 Jun 29;6:40. doi: 10.1038/s41378-020-0156-0 (PMC8433291; doi:10.1038/s41378-020-0156-0)
Supplement: Supplementary file 4 — Editorial summary [file 41378_2020_156_MOESM4_ESM.docx]

# *Microsystems & Nanoengineering*

Microfluidics: Surface tissue assays

A microfluidic device enables eight independent assays to be performed simultaneously on the surface of topologically irregular tissue sections. Current limitations for assays of tissue slices include, typically, a limited amount of tissue sample – limiting the range of assays that can be performed for a uniform sample – and sensitivity to the surface of the tissue, which might be inconsistent due to the spatially irregular composition of tissue. Here, a team from the Center for Research and Advanced Studies of the National Polytechnic Institute, Mexico, led by Jose Garcia-Cordero, has developed a microfluidic device that can perform assays on tissue sections with a wide range of topologies and thickness. The device consists of eight independent channels, enabling parallelization, and is demonstrated for tissue from an aorta, for both *in vitro* and *ex-vivo* assays.

Related article manuscript number: MICRONANO-00953R1

Article title: A versatile microfluidic device for multiple ex vivo/in vitro tissue assays unrestrained from tissue topography

Corresponding author and affiliation/s: Jose L. Garcia-Cordero, Centro de Investigación y de Estudios Avanzados del IPN, Unidad Monterrey, Apodaca, NL, Mexico

**About your Editorial Summary — please read**

**Before approving this Editorial Summary, please carefully check that (1) the summary text lists the correct author(s) and (2) the spelling and order of all author names and affiliations are correct.**

This **Editorial Summary** is based on your manuscript that was recently accepted for publication in *Microsystems & Nanoengineering*. It provides a non-specialist audience with a synopsis of your key research outcomes and conclusions. This value-added service provided by Springer Nature is designed to raise interest in your research across the broader community.

Springer Nature will publish the summary on the journal’s website, and it will be freely available under a under the CC BY licence (Creative Commons Attribution v4.0 International Licence) (see the journal website for details). We encourage you to re-use the summary to bring attention to your research; for example, you can host it on your own website and share it via social-networking platforms. Please attribute the summary to *Microsystems & Nanoengineering* and your article (e.g. by providing a link to your article) and do not make derivatives.

Please note that to maximise the usefulness of these summaries they must follow several stringent guidelines:
-- Spelling, punctuation and style are set according to *Nature* editorial guidelines. As this summary is aimed at non-expert readers, some concepts and technical terms will be simplified.
-- Total length must be no more than 135 words. It is likely that not all points in the paper will be covered.
-- The first sentence must be no more than 280 characters, including spaces, to allow use on microblogging sites.
-- The headline must consist of a brief generic subject identifier followed by a short description. No more than 10 words in total.

Please contact the editorial office ([mems_nano@mail.ie.ac.cn](mailto:mems_nano@mail.ie.ac.cn)) immediately with corrections should you find any factual errors in this Editorial Summary.
